# Comparative efficacy of neuromodulation techniques for Alzheimer’s disease: a systematic review and network meta-analysis of randomized controlled trials

**DOI:** 10.3389/fnins.2026.1890235

**Published:** 2026-09-11

**Authors:** Yuan Liu, Shiyu Liu, Huayu Yan, Xin Yang, Yabo Wu, Chang Liu, Yumin Xu

**Affiliations:** 1 Department of Encephalopathy, The First Affiliated Hospital of Henan University of Chinese Medicine, Zhengzhou, China; 2 The First Clinical Medical College of Henan University of Chinese Medicine, Zhengzhou, China; 3 Collaborative Innovation Center of Prevention and Treatment of Major Diseases by Chinese and Western Medicine, Zhengzhou, China

**Keywords:** Alzheimer’s disease, efficacy, network meta-analysis, neuromodulation techniques, prospect

## Abstract

**Background:**

Neuromodulation techniques (NMTs) have shown potential therapeutic value in Alzheimer’s disease (AD), but the comparative efficacy of different modalities remains unclear. This study aimed to systematically compare and rank available NMTs to identify their relative benefits across cognitive, functional, and neuropsychiatric outcomes.

**Objective:**

To evaluate and rank the efficacy of NMTs in improving cognitive function, activities of daily living (ADLs), and neuropsychiatric symptoms in AD patients, thereby providing evidence-based guidance for clinical intervention strategies.

**Methods:**

English-language literature was systematically searched in PubMed, Web of Science, Cochrane Library, and EMBASE from database inception to February 2026. Inclusion criteria were randomized controlled trials (RCTs) of AD patients aged ≥18 years. Interventions included repetitive transcranial magnetic stimulation (rTMS), transcranial alternating current stimulation (tACS), transcranial direct current stimulation (tDCS), photobiomodulation (PBM), transcutaneous electrical stimulation (TES), transcranial ultrasound stimulation (TUS), transcranial pulse stimulation (TPS), deep brain stimulation (DBS), and sham stimulation (Sham). Outcome measures included Mini-Mental State Examination (MMSE), Montreal Cognitive Assessment (MoCA), Alzheimer’ s Disease Assessment Scale-Cognitive Subscale (ADAS-Cog), Alzheimer’ s Disease Cooperative Study-Activities of Daily Living (ADCS-ADL), Boston Naming Test (BNT), Neuropsychiatric Inventory (NPI), and Geriatric Depression Scale (GDS). Data extraction and risk-of-bias assessment followed Cochrane guidelines. Network meta-analysis (NMA) was conducted in Stata 17.0, with effect sizes ranked by the surface under the cumulative ranking curve (SUCRA).

**Results:**

A total of 36 RCTs involving 1,445 patients were included in the analysis. Compared with Sham, TPS significantly improved MMSE (MD = 3.92, 95% CI: 1.57 to 6.35; SUCRA = 97.50%), MoCA (MD = 4.99, 95% CI: 2.26 to 7.72; SUCRA = 69.25%), and BNT scores (MD = 4.02, 95% CI: 1.81 to 6.23; SUCRA = 81.8%). rTMS also showed favorable effects on ADAS-Cog scores (MD = −11.10, 95% CI: −14.51 to −7.71; SUCRA = 91.00%). Regarding activities of daily living, TES ranked highest on the ADCS-ADL scale (SUCRA = 71.00%). In terms of neuropsychiatric symptoms, TPS achieved the highest ranking for improving NPI scores (SUCRA = 92.3%), whereas tACS showed the best performance on GDS scores (SUCRA = 69.5%).

**Conclusion:**

Neuromodulation interventions demonstrated domain-specific benefits in AD, with no single approach showing universal superiority. TPS demonstrated the greatest potential for improving global cognitive outcomes, while rTMS showed advantages in ADAS-Cog performance. Nevertheless, limited sample sizes, heterogeneous stimulation protocols, and insufficient long-term evidence restrict the generalizability of current findings. Future multicenter RCTs with standardized protocols and prolonged follow-up are needed to confirm these results and guide clinical implementation.

**Systematic review registration:**

PROSPERO, CRD420261364193.

## 1 Introduction

Alzheimer’s disease (AD), the most common form of dementia, is a progressive neurodegenerative disorder characterized clinically by cognitive decline and impaired daily functioning, and pathologically by amyloid-β plaques (Aβ), neurofibrillary tangles (NFT), neuroinflammation, synaptic dysfunction, and neuronal loss (Soria Lopez et al., 2019). According to a projection study based on the Global Burden of Disease (GBD) database, approximately 57.4 million individuals worldwide were living with dementia in 2019, and this number is expected to increase substantially to 152.8 million by 2050 owing to population growth and aging (GBD 2019 Dementia Forecasting Collaborators, 2022). Current treatments include pharmacological interventions, encompassing disease-modifying therapies that aim to slow progression but face limitations, and symptomatic therapies that alleviate cognitive and behavioral deficits without halting disease (Liu et al., 2024; Imbimbo et al., 2025). Consequently, nonpharmacological approaches, especially neuromodulation techniques (NMTs), have gained increasing attention for their potential to improve cognition and functional outcomes, though further research is needed to establish their efficacy and optimize treatment protocols (Cummings, 2021).

In recent years, advances in the understanding of AD pathophysiology and biotechnology have promoted the development of disease-modifying therapies (DMTs). By targeting key pathological processes, including Aβ deposition, tau hyperphosphorylation, neuroinflammation, and synaptic dysfunction, DMTs aim to slow disease progression (Cummings and Fox, 2017). Anti-Aβ monoclonal antibodies, such as lecanemab and donanemab, have provided new therapeutic options for patients with early-stage AD and demonstrated modest cognitive benefits. However, meta-analyses have revealed safety concerns associated with anti-Aβ therapies, particularly amyloid-related imaging abnormalities (ARIA), with an overall risk approximately 4.35-fold higher than placebo, including increased risks of ARIA-E and ARIA-H(Wang et al., 2025b). Meanwhile, DMTs targeting other pathological mechanisms, such as tau pathology, neuroinflammation, and synaptic dysfunction, remain under investigation (Cummings et al., 2024). Therefore, safe and effective adjunctive therapies are urgently needed. Neuromodulation has emerged as a promising complementary strategy to pharmacological DMTs by enhancing cognitive function and neuroplasticity. However, its comparative efficacy and optimal stimulation protocols remain unclear.

NMTs modulate abnormal neural activity by targeting specific neural nuclei or network nodes via invasive or non-invasive approaches, employing electrical, magnetic, acoustic, optical, or chemical stimulation (Li et al., 2023). These approaches are generally classified into invasive brain stimulation (IBS) and non-invasive brain stimulation (NIBS). IBS primarily include deep brain stimulation (DBS), and spinal cord stimulation (SCS), as well as other techniques. Whereas NIBS include repetitive transcranial magnetic stimulation (rTMS), transcranial alternating current stimulation (tACS), transcranial direct current stimulation (tDCS), photobiomodulation (PBM), transcutaneous electrical stimulation (TES), transcranial ultrasound stimulation (TUS), transcranial pulse stimulation (TPS). Currently, rTMS, tDCS, tACS, PBM, TES, TUS, DBS, and TPS have advanced substantially and are increasingly recognized as safe and promising therapeutic strategies for AD, and this review provides a comparative evaluation of their efficacy.

rTMS, a widely used non-invasive NMT, generates rapidly changing magnetic fields via brief, high-intensity electrical currents through a stimulation coil, which penetrate the scalp and skull to induce localized electric fields in targeted cortical regions (Vucic et al., 2023). In the early and intermediate stages of AD, rTMS has been reported to modulate neural network plasticity and improve cognitive performance, particularly in memory and language domains (Budak et al., 2023).

tACS is an emerging NIBS that selectively modulates neural oscillatory activity. By entraining neuronal synchronization and brain network rhythms, tACS regulates neural activity and synaptic plasticity and may offer advantages over rTMS and tDCS in terms of safety and reduced susceptibility to external interference (Wischnewski et al., 2023). Previous clinical studies have demonstrated that 40 Hz tACS can improve short-term memory and language learning performance in patients with AD (Benussi et al., 2022).

tDCS uses two or more electrode pads to deliver weak electrical currents to the target brain regions, thereby modulating the transmembrane potential of neurons(Tariq et al., 2023), and significantly improves cognitive function in AD by modulating cortical excitability, enhancing synaptic plasticity, and regulating cholinergic and dopaminergic pathways, with immediate and sustained benefits in Mini-Mental State Examination (MMSE) scores (Prathum et al., 2025).

TPS activates mechanosensitive neuronal ion channels to induce controlled calcium influx and membrane depolarization without causing tissue heating, cavitation, or blood–brain barrier disruption. This calcium signaling further upregulates c-Fos expression in the dentate gyrus (DG) of the hippocampus, selectively activating memory-related circuits impaired in AD and improving cognition and neuropsychiatric symptoms (Karakatsani et al., 2025).

DBS involves stereotactic implantation of electrodes into specific intracerebral targets to deliver electrical pulses that modulate neuronal activity (Okun, 2014), and has also been shown to attenuate neuroinflammation, reduce Aβ deposition and neuronal loss in the hippocampus and cortex, and suppress microglial and astrocytic activation, with therapeutic efficacy influenced by stimulation parameters such as frequency, pulse width, current intensity, voltage, cycle, and duration (Leplus et al., 2019; Yang et al., 2021).

Evidence on NMTs for AD remains fragmented, as traditional pairwise meta-analyses usually compare only individual neuromodulation techniques and rarely integrate direct and indirect evidence across multiple modalities. This systematic review and NMA evaluates the efficacy of eight interventions, including rTMS, tACS, tDCS, PBM, TES, TUS, DBS, and TPS, compared with sham stimulation (Sham). The aim of this study is to evaluate and compare the efficacy of different NMTs in improving cognitive outcomes in patients with AD.

## 2 Methods

### 2.1 Study design and registration

This study was conducted in accordance with the PRISMA guidelines and was registered with the International Prospective Register of Systematic Reviews (No. CRD420261364193) (Elsman et al., 2024).

### 2.2 Literature search

We searched PubMed, Embase, the Cochrane Central Register of Controlled Trials, and Web of Science to identify English-language RCTs published from database inception through February 2026. The search strategy was carefully designed by integrating MeSH terms with free-text terms and was optimized specifically for the unique attributes of each record. The search queries were systematically divided into three major categories: NMTs, AD, and RCTs. The NMTs included in the search were rTMS, tACS, tDCS, PBM, TES, TUS, DBS, TPS, and Sham. Supplementary Table 1 presents an example of the complete search strategy.

### 2.3 Screening criteria

The included studies were full-text, English-language RCTs that met the following criteria: (1) participants were diagnosed with AD at any stage of the disease; (2) the trials investigated eight NMTs (rTMS, tACS, tDCS, PBM, TES, TUS, DBS, and TPS), either alone or in combination, compared with Sham; (3) the treatment dose was clearly defined and within the therapeutic range; (4) the criteria for disease severity classification were appropriate; (5) outcomes reported included at least one of the following measures: MMSE, Montreal Cognitive Assessment (MoCA), Alzheimer’ s Disease Assessment Scale-Cognitive Subscale (ADAS-Cog), Alzheimer’ s Disease Cooperative Study-Activities of Daily Living (ADCS-ADL), Boston Naming Test (BNT), Neuropsychiatric Inventory (NPI), and Geriatric Depression Scale (GDS).

Exclusion criteria were as follows: (1) investigations that were not RCTs, including experimental studies, meta-analyses, systematic reviews, conference abstracts, case reports, editorials, and other non-primary research; (2) studies not directly addressing the research question; (3) full-text articles that did not meet the PICOS criteria (population, intervention, comparator, outcomes, and study design).

### 2.4 Data extraction

Two researchers (YL and SL) independently completed the literature screening and data extraction using EndNote reference management software. First, duplicate records were removed from the imported literature. The studies were then screened in strict accordance with the predefined inclusion and exclusion criteria, and the extracted data were cross-checked independently. In cases of disagreement, a third researcher (HY) participated in the discussion, and the issue was resolved through consultation. For the studies ultimately included, basic information was extracted using Excel software, including first author, year of publication, sample size, age, intervention measures, treatment duration, and outcome indicators.

### 2.5 Quality assessment

Two independent reviewers (YL and SL) assessed the included studies using the revised Cochrane risk-of-bias tool for randomized trials (RoB2.0). Any disagreements were resolved through consensus or consultation with a third reviewer (HY). The assessment considered five domains: bias arising from the randomization process, bias due to deviations from intended interventions, bias due to missing outcome data, bias in outcome measurement, and bias resulting from selective reporting of results. An overall risk-of-bias judgment was then assigned to each study.

### 2.6 Statistical analysis

The revised Cochrane risk of bias assessment tool version 2.0 (RoB 2.0) was used for literature quality evaluation. Stata 17 was used to perform traditional meta-analysis and network meta-analysis based on a frequentist framework for the included literature. Heterogeneity was assessed using *I*^2^ statistic. A random-effects model was used if *I*^2^ > 50% or *p* ≤ 0.10; otherwise, a fixed-effects model was adopted. For outcome indicators, if the variable was categorical, the odds ratio (OR) was used for calculation; if it was a continuous variable, the mean difference (MD) was used; both were expressed as effect sizes with their 95% confidence intervals (CI). No closed loops were identified in the evidence network for any outcome, precluding formal inconsistency assessment using node-splitting methods; therefore, all network meta-analyses were performed under the assumption of consistency. The surface under the cumulative ranking curve (SUCRA) was used to rank the outcome indicators of each intervention.

## 3 Results

### 3.1 Literature search results

**Figure 1:**
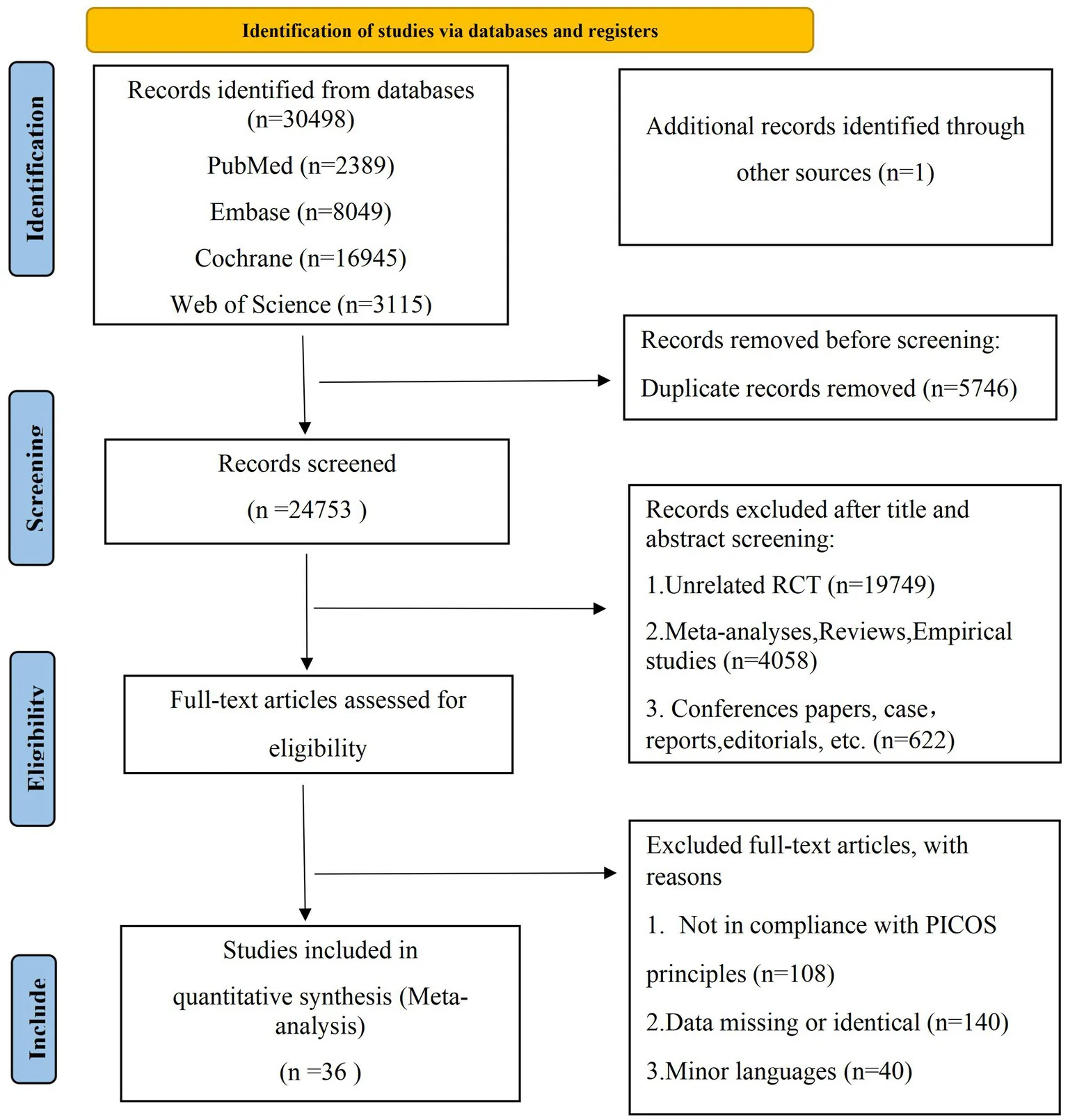
The flow diagram of the literature screening process.

A total of 30,499 articles were retrieved, including 5,746 duplicates. After screening titles and abstracts, 24,717 were excluded for non-RCT design, inconsistent interventions, or being reviews, commentaries, animal studies, or other irrelevant literature. Full-text review led to the exclusion of 108 articles for methodological issues and 180 for missing data or non-English publication. Ultimately, 36 articles were included. The complete screening process is shown in Figure 1.

### 3.2 Basic information for the included studies

A total of 36 RCTs were included, comprising 1,445 patients, including 778 in the treatment groups and 667 in the control groups. Eight neuromodulation techniques were involved: rTMS in 18 studies, tACS in 2 studies, tDCS in 6 studies, PBM in 5 studies, TES in 1 study, TUS in 1 study, DBS in 1 study, and TPS in 2 studies. Sham was the most common control condition. The comprehensive baseline characteristics and design elements of the studies included in this network meta-analysis are presented in the Table 1.

**Table 1:** Basic information of included studies.

Study	Country	Intervention	Sample size		Gender (M/F)		Mean age (year)		MMSE(baseline)		Session duration/day	Sessions per week/Total sessions	Outcome
			EG	CG	EG	CG	EG	CG	EG	CG			
Wu et al. (2015)	China	rTMS	26	26	15/11	10/16	71.9 ± 4.8	71.4 ± 4.9	15.2 ± 3.1	15.3 ± 3.1		5/20	F1
		(20 Hz)											
Li et al. (2021)	China	rTMS	38	37	24/14	20/17	64.5 ± 7.8	65.9 ± 8.4	15.9 ± 4.1	16.1 ± 4.2	20 min	5/30	F1, F2
		(20 Hz)											
Zhao et al. (2024) NBKNYY-2021-LC-40	China	rTMS (1/10 Hz)	16	17	7/9	7/10	72.6 ± 6.7	73.7 ± 3.8	17.0 ± 3.0	17.6 ± 4.1	30 min	5/20	F2, F3, F4
Yao et al. (2022) ChiCTR2100043362	China	rTMS (5 Hz)	12	15	6/6	8/7	67.6 ± 7.8	63.8 ± 6.8	18.4 ± 5.0	19.8 ± 4.3	20 min	5/20	F1, F2, F5, F6
Jung et al. (2024) NCT04260724	South Korea	rTMS (20 Hz)	12	18			73.1 ± 8.2	72.5 ± 7.5	22.5 ± 3.6	23.9 ± 3.5	20 min	5/20	F1, F2, F3, F5
Koch et al. (2022) NCT05454540	Italy	rTMS (20 Hz)	14	18			71.2 ± 5.1	74 ± 5.1	21.9 ± 3.07	20.9 ± 2.8	20 min	5/10	F1, F2, F3, F4
Ahmed et al. (2012)	Egypt	rTMS (1/20 Hz)	11	21				68.6 ± 6.7 65.9 ± 5.9	15.4 ± 2.8	15.5 ± 3.5 18.4 ± 2.7		5/5	F2, F7
Bagattini et al. (2020)	Italy	rTMS+CT	23	27	12/11	17/10	73.3 ± 1.0	73.5 ± 4.9	22.7 ± 0.5	23.6 ± 3.0	25 min	5/20	F2, F7
		(20 Hz)											
Zhang et al. (2019) ChiCTR-INR-16009227	China	TMS + CT (10 Hz)	13	15		12/3		69.0 ± 8.1	19.8 ± 5.1	20.5 ± 4.1	20 min	5/20	F1, F2, F4
Chen et al. (2023)	China	rTMS	6	18	4/2	7/11	67.1 ± 8.7	66.6 ± 7.4	29.3 ± 1.0	25.0 ± 4.9		5/20	F2, F5, F6
		(20 Hz)											
Vecchio et al. (2022)	United States	rTMS+CT (10 Hz)	17	30	10/7	14/16	72.2 ± 2.2	71.0 ± 1.2	20.6 ± 0.6	22.9 ± 0.5		5/30	F1
Wei et al. (2022) MR-33-20-004217	China	rTMS (10 Hz)	27	29	7/20	9/20	71.6 ± 7.1	70.0 ± 8.6	13.7 ± 7.1	14.4 ± 6.9	20 min	5/10	F2, F3
Koch et al. (2022) NCT03778151	Italy	rTMS (20 Hz)	12	18			69.8 ± 9.1	69.8 ± 9.1	22.5 ± 3.6	23.9 ± 3.5		5/20	F1, F2, F5
Jia et al. (2021) MR33-20-004217	China	rTMS (10 Hz)	34	35	11/23	25/10	73.4 ± 7.7	71.4 ± 8.8	15.6 ± 6.4	15.7 ± 5.6		5/10	F2, F5
Saitoh et al. (2022) jRCTs052180226	Japan	rTMS (10 Hz)	12	28	4/8	11/17	75.8	76.2	18.6 ± 4.9	18.2 ± 4.8		4/8	F1, F2, F4
Zhao et al. (2017)	China	rTMS	13	17	6/7	7/10	71.4 ± 5.2	69.3 ± 5.8	22.8 ± 2.3	22.2 ± 2.8	60 min	5/30	F1, F2
		(20 Hz)											
Koch et al. (2018)	Italy	rTMS	14	14	7/7	7/7	70.0 ± 5.1	70.0 ± 5.1	26.7 ± 2.6	26.9 ± 1.9	20 min	5/10	F2
		(20 Hz)											
Lee et al. (2016)	South Korea	rTMS+CT	8	18	3/5	8/10	70.3 ± 4.8	72.1 ± 7.6	22.7 ± 2.4	22.3 ± 2.8	60 min	5/30	F1, F2
		(10 Hz)											
Zou et al. (2022)	China	PBM	27	34	12/15	16/18	73.0 ± 9.3	75.9 ± 9.4	8.7 ± 4.5	9.1 ± 4.5	30 min	7/28	F4
		14000Lux											
Figueiro et al. (2019) NCT01816152	United States	PBM 350–750 Lux	46	46				85.3 ± 7.7		16.5 ± 5.0	12 h	7/98	F3
Chao (2019)	United States	PBM	4	4	2/2	1/3	79.0 ± 5.9	80.5 ± 6.5	22.3 ± 1.3	19.5 ± 7.0	20 min	3/36	F1, F4
		810 nm											
Razzaghi et al. (2024) IRCT20111121008146N35	Iran	PBM 150 ± 10 mW/cm^2^	7	6	5/2	5/1	75.8 ± 7.1	74.6 ± 4.4			20 min	6/72	F5
Blivet et al. (2022) NCT03672474	France	PBM 850 nm	26	27	10/16	12/15	73.7 ± 6.4	72.4 ± 7.0	20.2 ± 3.5	20.5 ± 3.6	25 min	5/40	F1, F2
van Dijk et al. (2005)	Netherlands	TES 160 Hz	30	32	22/8	17/15	72.5 ± 8.2	71.0 ± 7.8	14.7 ± 7.2	15.7 ± 6.8	30 min	7/42	F3, F7
Olazarán et al. (2013)	Spain	tACS 10.5 Hz	14	17	2/12	5/12	85.4 ± 1.2	84.2 ± 2.1	7.1 ± 2.5	8.5 ± 2.4	2 h		F2, F3, F4
Tang et al. (2024) NCT03920826	China	tACS 40 Hz	23	23	9/14	7/16	63.7 ± 6.0	65.8 ± 5.2	20.7 ± 3.0	19.4 ± 3.3	2 h	7/15	F1, F2, F4, F5, F6, F7
Im et al. (2019)	South Korea	tDCS	7	11	2/5	1/10	74.9 ± 5.0	71.9 ± 9.2	22.1 ± 4.6	20.1 ± 3.8	30 min	7/180	F2, F6
		2 mA											
Gangemi et al. (2021)	Italy	tDCS 2 mA	13	13			69.0 ± 3.1	67.5 ± 2.8	16.0 ± 1.7	15.1 ± 2.4	20 min	7/10	F2
Martorella et al. (2023) NCT04457973	United States	tDCS 2 mA	20	20	5/15	6/14	71.9 ± 9.2	74.1 ± 6.3			20 min	5/5	F4
Satorres et al. (2022)	Spain	tDCS 2 mA	16	17	8/8	9/8	73.4 ± 6.2	76.6 ± 5.7	22.9 ± 3.9	23.8 ± 3.2	20 min	7/10	F2
Khedr et al. (2019) NCT03313518	Egypt	tDCS 2 mA	21	23	13/8	10/13	65.2 ± 4.5	64.2 ± 3.6	18.7 ± 12.6	15.4 ± 10.0	20 min	5/10	F2
Meléndez et al. (2023)	Spain	tDCS 2 mA	9	9	3/6	4/5	75.4 ± 4.4	73.7 ± 3.8	18.8 ± 2.1	18.1 ± 1.5	20 min	5/5	F2
Wu et al. (2022) NCT03612622	China	TPS 1800 pulses	23	24	11/12	10/14	66.3 ± 7.9	66.4 ± 8.2	21.7 ± 4.6	20.5 ± 4.6		7/14	F2, F3, F5
Wu et al. (2024) NCT04754152	China	TPS 1800 pulses	22	20	7/15	6/14	65.3 ± 7.3	66.8 ± 8.8	21.1 ± 3.9	21.3 ± 5.4		7/14	F2, F3, F4, F5, F6
Leoutsakos et al. (2018) NCT01608061	United States and Canada	DBS	21	21								12 months	F1, F4
Matt et al. (2025) NCT03770182	Austria	TUS (5 Hz)	30	30			70.6 ± 8.1	70.6 ± 8.1				3/6	F1, F4, F7

EG, experimental group; CG, control group CT, Cognitive Training; MMSE, Mini-Mental State Examination; MoCA, Montreal Cognitive Assessment; ADAS-Cog, Alzheimer’s Disease Assessment Scale-Cognitive Subscale; ADCS-ADL, Alzheimer’s Disease Cooperative Study-Activities of Daily Living; BNT, Boston Naming Test; NPI, Neuropsychiatric Inventory; GDS, Geriatric Depression Scale; FI: ADAS-Cog; F2: MMSE; F3: ADCS-ADL; F4: NPI; F5: MoCA; F6: BNT; F7: GDS.

### 3.3 Risk of bias

**Figure 2:**
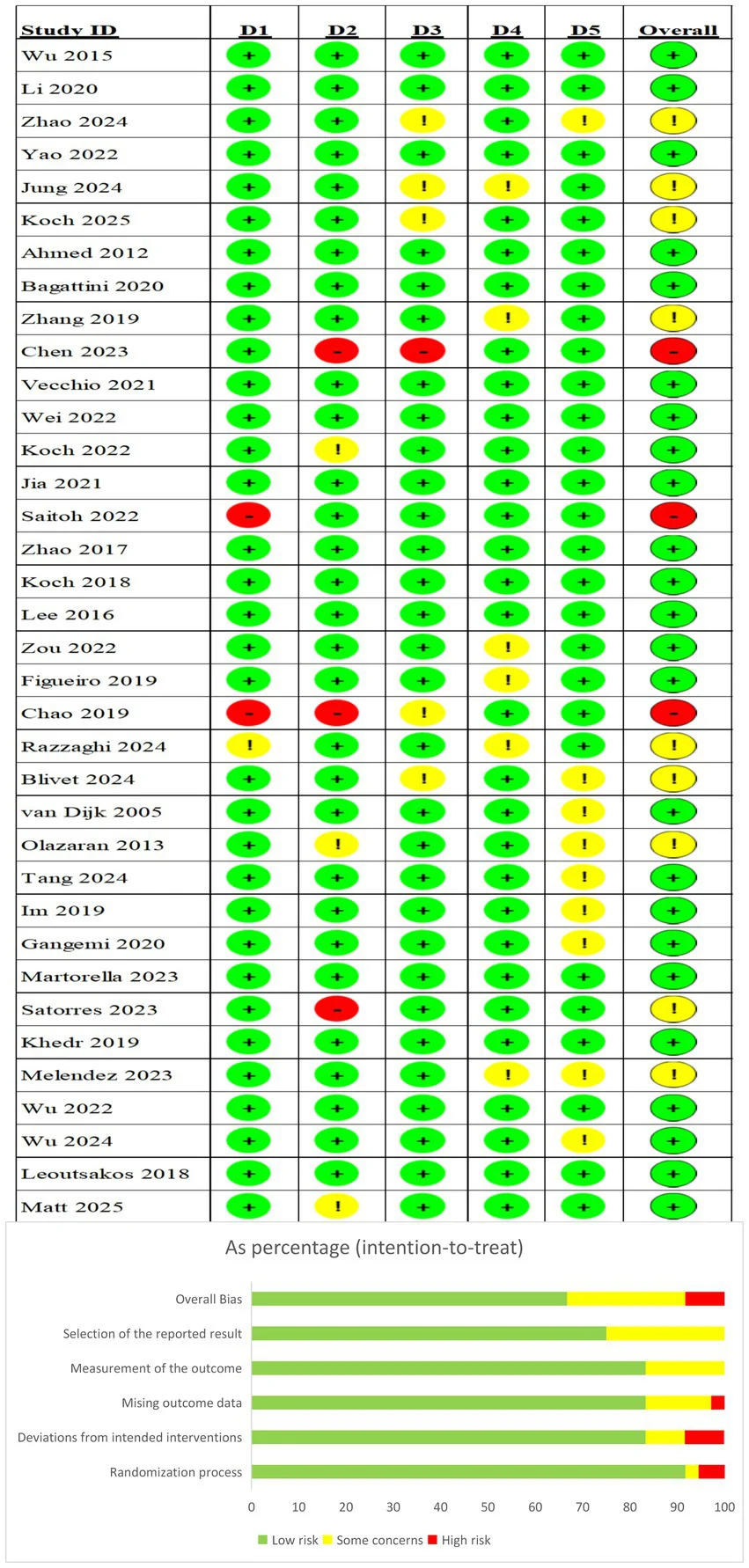
Risk of bias summary and risk of bias graph for included studies. D1: Randomisation process; D2: Deviations from intended interventions; D3: Missing outcome data; D4: Measurement of the outcome; D5: Selection of the reported result: Low risk: Some concerns: High risk.

The methodological quality of the 36 included studies was systematically assessed using the revised Cochrane Risk of Bias tool (RoB 2.0). Among the studies, 94% had adequately documented randomization (“low”selection bias), while 6% had serious deficiencies. Intervention adherence was high in 83.3% of patients, with 8% showing moderate concerns and 8.7% substantial deviations. One study had severe missing data, and four had incomplete records. Five studies (13.9%) lacked clearly defined measurement protocols, indicating potential detection bias. Selective reporting raised moderate concerns in 25% of studies. Overall, 8.3% of studies were rated high risk and 25% moderate risk. Risk distributions are presented in Figure 2.

## 4 Network meta-analysis

**Figure 3:**
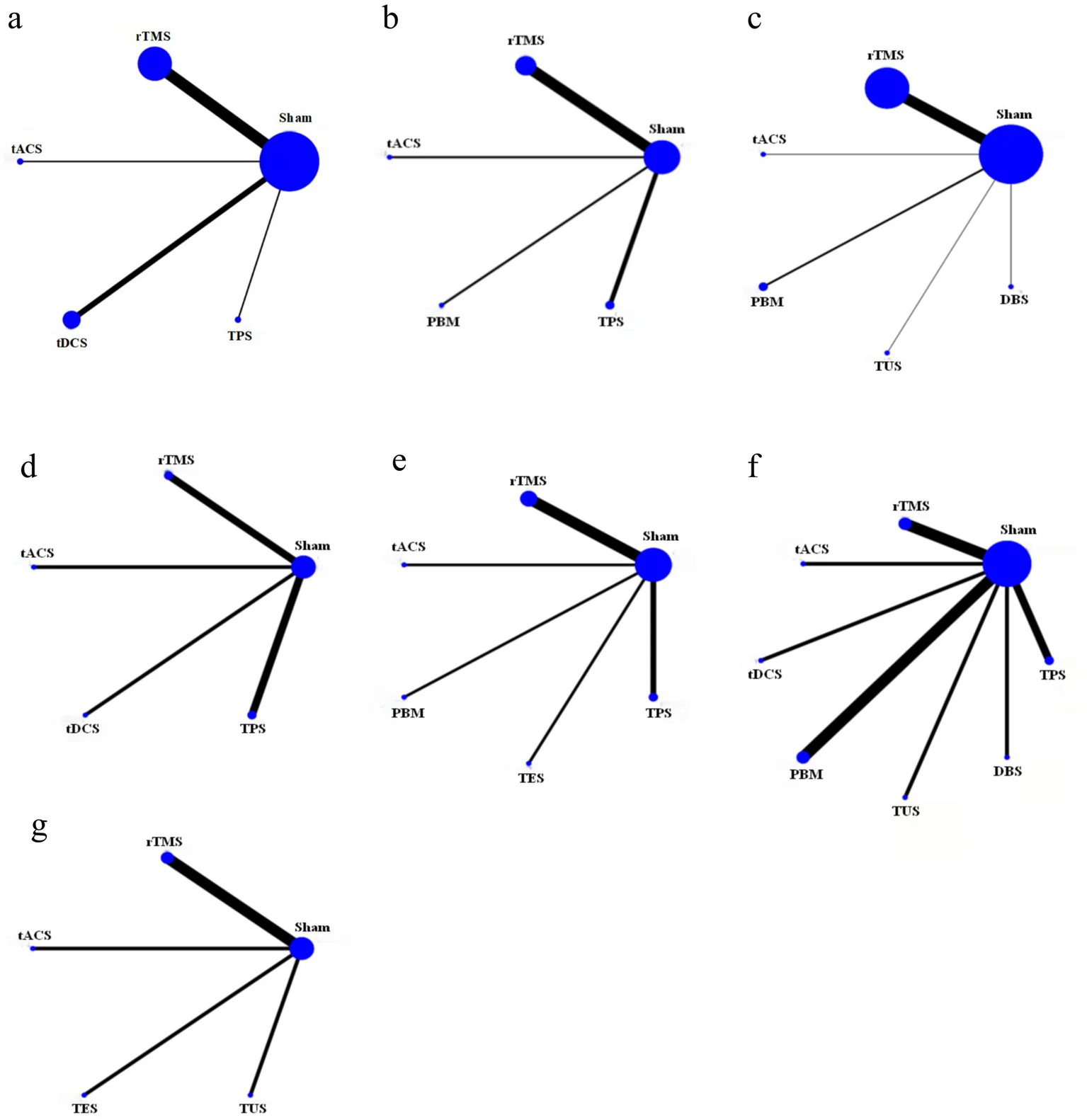
Network diagrams illustrating the comparative effectiveness of various NMTs for Alzheimer’s. Panels **(a–g)** show different treatment networks, with Sham as a common comparator. **(a)** Mini-Mental State Examination (MMSE), **(b)** Montreal Cognitive Assessment (MoCA), **(c)** Alzheimer’s Disease Assessment Scale-Cognitive Subscale (ADAS-Cog), **(d)** Alzheimer’s Disease Cooperative Study-Activities of Daily Living (ADCS-ADL), **(e)** Boston Naming Test (BNT), **(f)** Neuropsychiatric Inventory (NPI), **(g)** Geriatric Depression Scale (GDS).

The Network meta-analysis (NMA) is shown in Figure 3, and the detailed NMA results can be found in Supplementary Figure 1.

### 4.1 Cognitive function outcomes

#### 4.1.1 Changes in MMSE

**Figure 4:**
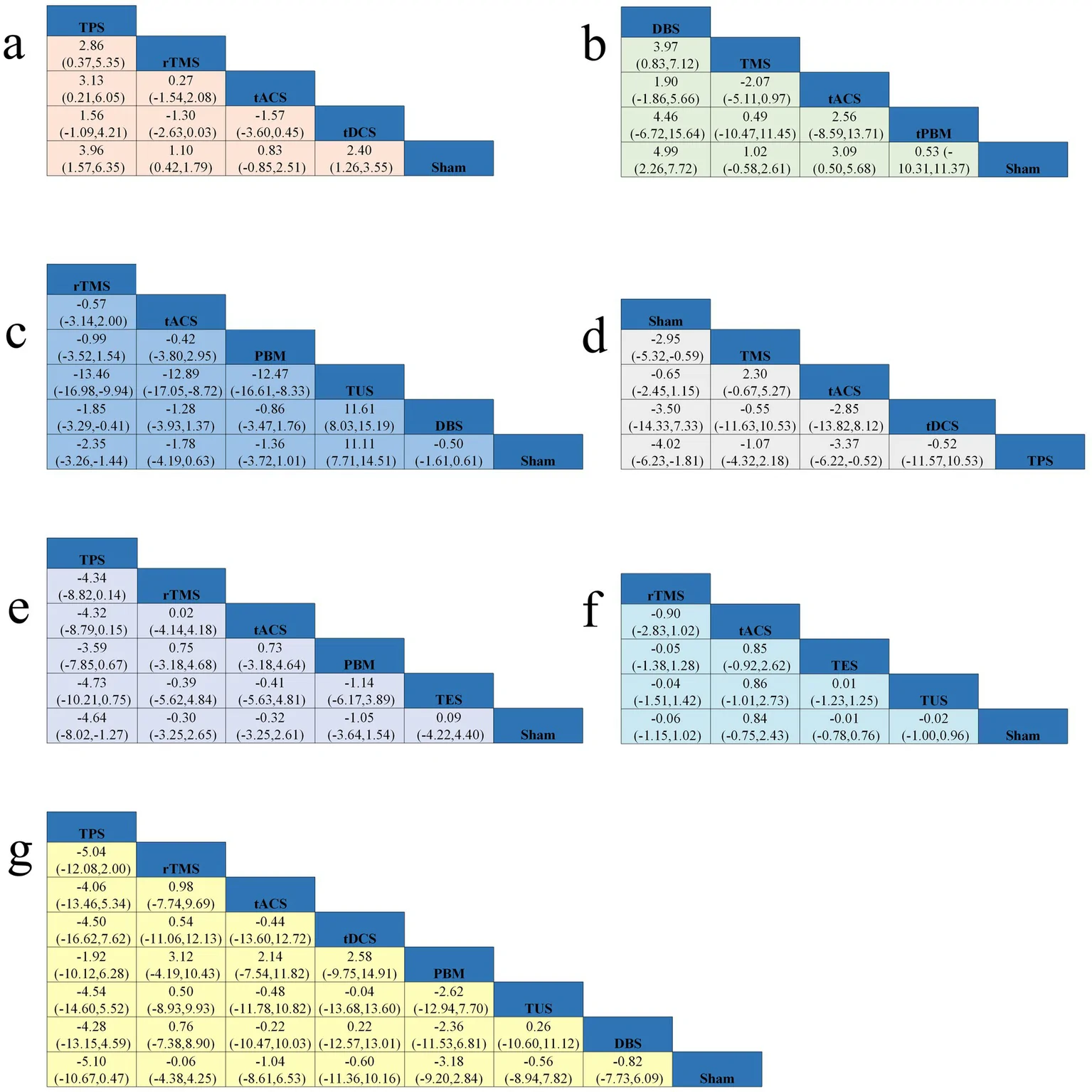
The results of the network meta-analysis. Note: MMSE, Mini-Mental State Examination; MoCA, Montreal Cognitive Assessment; ADAS-Cog, Alzheimer’s Disease Assessment Scale-Cognitive Subscale; ADCS-ADL, Alzheimer’s Disease Cooperative Study-Activities of Daily Living; BNT, Boston Naming Test; NPI, Neuropsychiatric Inventory; GDS, Geriatric Depression Scale **(a)** MMSE; **(b)** MoCA; **(c)** ADAS-Cog; **(d)** BNT; **(e)** ADCS-ADL; **(f)** GDS; **(g)** NPI.

**Figure 5:**
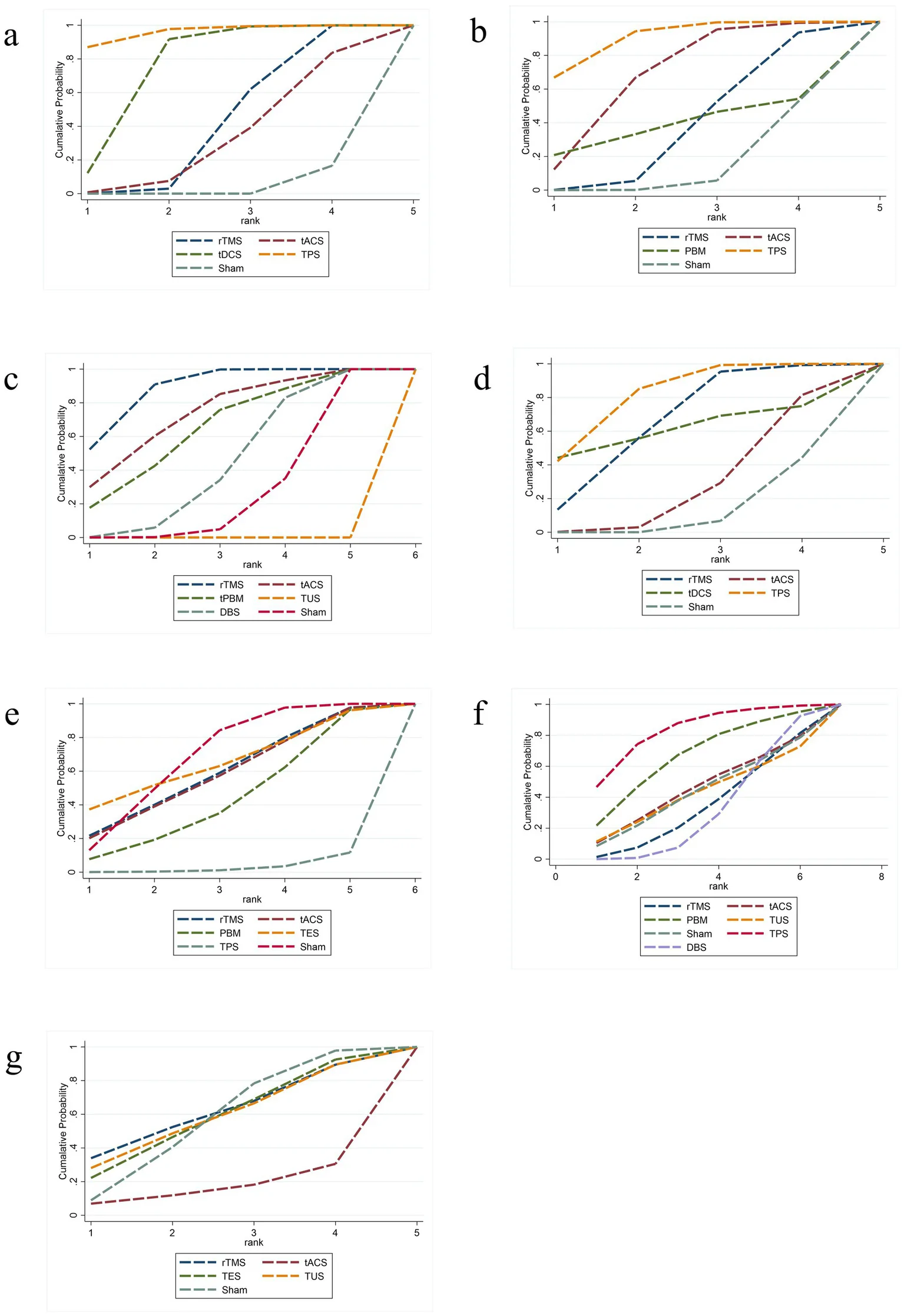
The results of SUCRA. **(a)** Mini-Mental State Examination (MMSE), **(b)** Montreal Cognitive Assessment (MoCA), **(c)** Alzheimer’s Disease Assessment Scale-Cognitive Subscale (ADAS-Cog), **(d)** Alzheimer’s Disease Cooperative Study-Activities of Daily Living (ADCS-ADL), **(e)** Boston Naming Test (BNT), **(f)** Neuropsychiatric Inventory (NPI), **(g)** Geriatric Depression Scale (GDS).

The analysis of MMSE scores included data from 25 RCTs involving four interventions, with all participants being patients with mild to moderate AD. The NMA results indicated that TPS, tACS, and tDCS were significantly superior to Sham in improving MMSE performance, whereas rTMS did not reach statistical significance. SUCRA analysis ranked the interventions as follows: TPS (97.50%), tDCS (53.80%), tACS (19.3%), and rTMS (26.3%). (Figures 4a, 5a).

#### 4.1.2 Changes in MoCA

This NMA included 9 RCTs with a total of 328 participants, assessing the effects of four interventions: rTMS, tACS, PBM, and TPS on MoCA scores. NMA revealed a significant cognitive benefit of TPS compared with Sham on MoCA scores (MD = 4.99, 95% CI: 2.26–7.72). SUCRA-based hierarchical ranking indicated that TPS achieved the highest probability of being the most effective intervention for MoCA improvement (SUCRA = 69.25%, PrBest = 67%, mean rank = 2.2), whereas PBM showed the least favorable ranking (SUCRA = 23.38%, mean rank = 4.1). The cumulative ranking curves (Figure 5b) further supported the observed efficacy ranking pattern among interventions.

#### 4.1.3 Changes in ADAS-cog

The ADAS-Cog analysis incorporated 16 articles, covering five interventions (rTMS, tDCS, PBM, TUS, and DBS) and involving 617 participants, assessing changes in ADAS-Cog scores from baseline. Compared with Sham, rTMS significantly improved cognitive function (MD − 11.1, 95% CI − 14.51, −7.71), whereas other interventions did not reach statistical significance. TUS showed the poorest efficacy. Interestingly, the Sham outperformed some interventions, suggesting a potential placebo effect or limitations in study design. Based on cumulative probability analysis, rTMS (SUCRA: 91.00%), tDCS (SUCRA: 78.00%), and PBM (SUCRA: 67.00%) were identified as the three most effective interventions for improving ADAS-Cog scores. (Figures 4c, 5c).

#### 4.1.4 Changes in BNT

Data on changes in BNT scores were derived from six RCTs involving four interventions (rTMS, tACS, tDCS, and TPS) and a total of 204 participants, with lower scores indicating more severe cognitive impairment. Compared with Sham, TPS (MD 4.02, 95% CI 1.81 to 6.23) and rTMS (MD 2.95, 95% CI 0.59 to 5.32) significantly improved BNT scores. TPS achieved the highest ranking, with a SUCRA value of 81.8%, a probability of being the best treatment (PrBest) of 42.0%, and a mean rank of 1.7, indicating that it was the most effective intervention for mitigating AD-related cognitive decline. In contrast, tACS had the lowest SUCRA value (28.5%) and the highest mean rank (3.8), suggesting limited therapeutic benefit relative to the other interventions. The cumulative probability curves further confirmed the efficacy advantage of TPS for improving BNT scores (Figures 4d, 5d).

### 4.2 Functional and global outcomes

#### 4.2.1 Changes in ADCS-ADL

A total of nine studies reported ADL outcomes, involving four active NMTs (rTMS, tACS, PBM, and TES) and Sham. The NMA generated 15 pairwise comparisons, of which only one showed statistical significance. Sham demonstrated significantly better outcomes than TES, suggesting a potential placebo effect or methodological limitations. According to cumulative probability analysis, TES (SUCRA: 71.00%), rTMS (SUCRA: 59.00%), and tACS (SUCRA: 57.00%) ranked as the three most effective interventions for improving ADCS-ADL scores (Figures 4e, 5e).

### 4.3 Neuropsychiatric symptom outcomes

#### 4.3.1 Changes in NPI

Based on data from 12 RCTs, this study used NMA to evaluate the effects on NPI scores, involving seven active interventions and Sham. A total of 423 patients were included, with higher scores indicating greater severity of neuropsychiatric symptoms. The active interventions included rTMS, tACS, tDCS, PBM, TUS, DBS, and TPS. A total of 28 pairwise comparisons were generated. None of the interventions showed a statistically significant difference compared with Sham in reducing NPI scores. The SUCRA ranking was as follows: TPS (92.3%) > PBM (76.2%) > tACS (50.1%) > Sham (47.8%) > rTMS (45.2%) > TUS (42.9%) > DBS (40.5%), indicating that TPS had the greatest potential for reducing NPI scores (Figures 4g, 5f).

#### 4.3.2 Changes in GDS

When evaluating changes in GDS from baseline, six studies involving 246 participants were included, with higher scores indicating more severe depression. The analysis included rTMS, tACS, TES, TUS, and Sham. The NMA results showed that none of the active interventions demonstrated a statistically significant difference compared with Sham in reducing GDS scores. The SUCRA ranking was as follows: tACS (69.5%) > Sham (58.8%) > TES (45.8%) > TUS (38.0%) > rTMS (32.5%), with tACS ranking highest among the neuromodulation strategies (Figures 4f, 5g).

### 4.4 Heterogeneity analysis

Heterogeneity was assessed using the *I*^2^ statistic. Moderate to high heterogeneity was observed for ADCS-ADL (*I*^2^ = 59.9%), NPI (*I*^2^ = 71.3%), and ADAS-Cog (*I*^2^ = 71.8%) outcomes. In contrast, MMSE (*I*^2^ = 24.6%), MoCA (*I*^2^ = 22.9%), GDS (*I*^2^ = 0%), and BNT (*I*^2^ = 0%) showed low heterogeneity and good consistency across studies.

To explore potential sources of heterogeneity, subgroup analyses were conducted to examine whether stimulation duration and frequency influenced treatment effects. These analyses were performed to assess the robustness of the findings and to quantitatively compare differences in effect sizes associated with stimulation parameters.

#### 4.4.1 Subgroup analysis

ADCS-ADL results: Subgroup analysis indicated that variability in ADCS-ADL results was related to TPS intervention duration. Long-term TPS was associated with significantly poorer activities of daily living performance (MD = −1.12, 95% CI: −1.77 to −0.46), whereas the 14-day TPS intervention showed no statistically significant effect (MD = −0.23, 95% CI: −0.81 to 0.34).

NPI results: For NPI, between-study heterogeneity appeared to be mainly driven by differences in rTMS stimulation frequency. Specifically, 10 Hz rTMS was associated with significantly worse neuropsychiatric symptoms (MD = 1.99, 95% CI: 1.07 to 2.91), whereas 20 Hz rTMS (MD = −0.51, 95% CI: −1.22 to 0.20) and alternating 1/10 Hz stimulation (MD = −0.65, 95% CI: −1.35 to 0.05) demonstrated non-significant trends toward symptom improvement.

ADAS-Cog results: The substantial variability observed in ADAS-Cog results was mainly associated with differences in intervention protocols, and no statistically significant differences were detected among subgroups.

### 4.5 Publication bias

**Figure 6:**
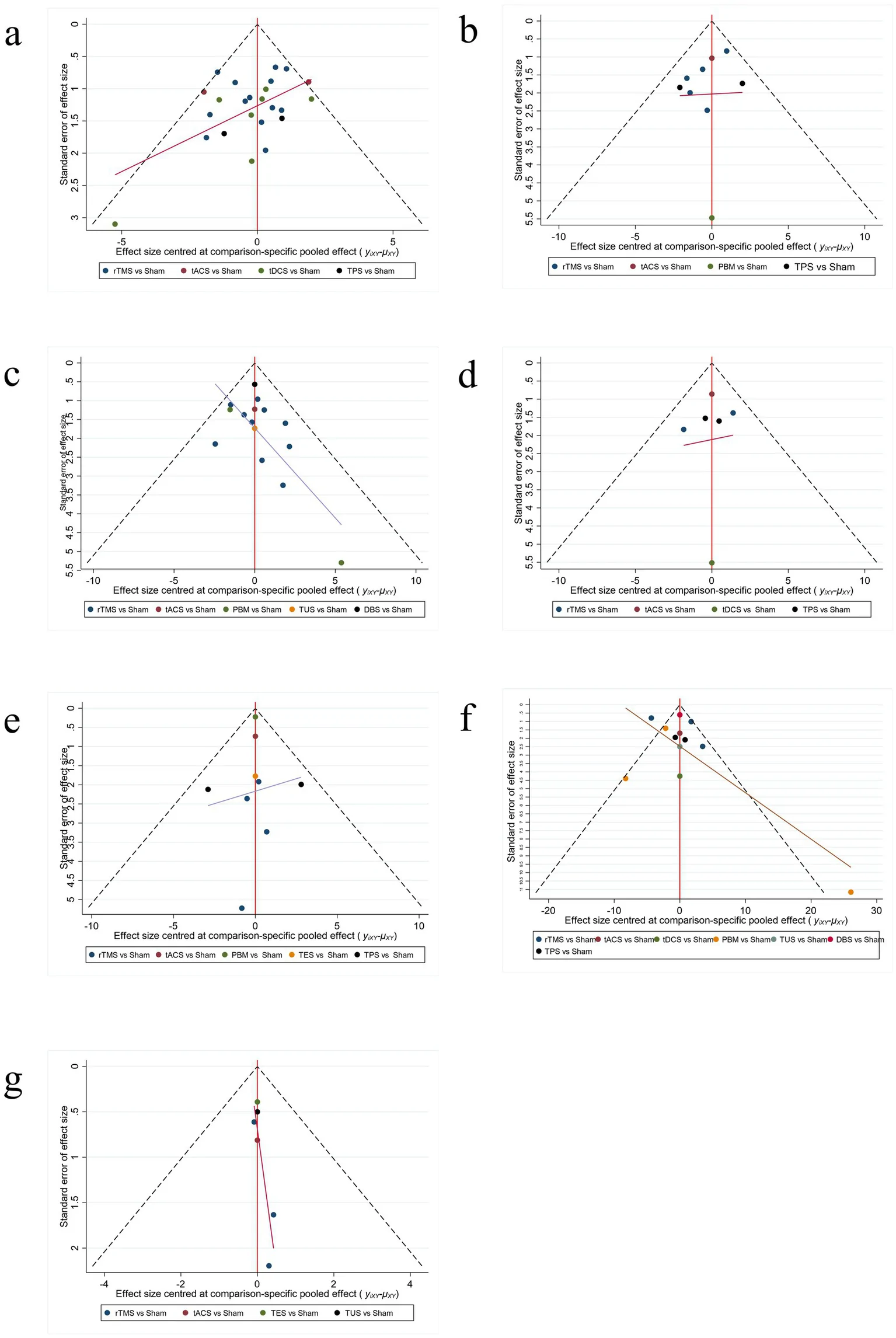
Funnel plots assessing publication bias for each outcome measure. **(a)** Mini-Mental State Examination (MMSE), **(b)** Montreal Cognitive Assessment (MoCA), **(c)** Alzheimer’s Disease Assessment Scale-Cognitive Subscale (ADAS-Cog), **(d)** Alzheimer’s Disease Cooperative Study-Activities of Daily Living (ADCS-ADL), **(e)** Boston Naming Test (BNT), **(f)** Neuropsychiatric Inventory (NPI), **(g)** Geriatric Depression Scale (GDS).

Funnel plots for all outcome indicators showed an approximately symmetrical distribution of studies on both sides of the vertical line, suggesting a low risk of publication bias (Figure 6).

## 5 Discussion

### 5.1 Study overview and Main findings

Consistent with the observed ranking pattern across the network meta-analysis (Figures 4, 5), different neuromodulation techniques demonstrated distinct therapeutic profiles rather than a universally superior treatment. TPS showed the greatest potential for improving global cognitive outcomes, whereas rTMS achieved the most favorable performance on ADAS-Cog. These outcome-specific differences suggest that different NMTs may provide complementary benefits across different clinical domains. Clinically, these findings support tailoring NMT selection to specific therapeutic targets rather than assuming that one modality is superior across all outcomes. However, these rankings should be interpreted in the context of the quantity and quality of the available evidence, as well as the heterogeneity in stimulation protocols. Larger multicenter RCTs with standardized stimulation parameters and consistent outcome measures are needed to validate the outcome-specific effects of TPS and rTMS and clarify their comparative clinical efficacy.

### 5.2 Differences in the efficacy of neuromodulation and evidence-based interpretation

The outcome-specific ranking observed in the present NMA may reflect fundamental differences in the mechanisms of action among NMTs. The favorable ranking of TPS is consistent with previous meta-analytic evidence suggesting greater benefits in memory-related outcomes compared with rTMS (Wang et al., 2026). One possible explanation is that TPS can deliver mechanical stimulation to relatively deep brain regions involved in memory processing, including the hippocampus (Karakatsani et al., 2025); However, this mechanistic explanation remains preliminary, and the apparent superiority of TPS should not be overinterpreted because the available evidence is derived from only two small randomized controlled trials, limiting direct comparisons and resulting in substantial uncertainty in the estimated treatment effects. In contrast, rTMS was supported by 18 RCTs, providing a comparatively larger evidence base. Previous meta-analytic evidence also showed that active rTMS significantly improved MMSE and MoCA scores and reduced ADAS-Cog scores in patients with AD and MCI compared with Sham (Wang et al., 2025a). Therefore, future research should prioritize larger, well-designed RCTs of TPS using standardized stimulation protocols and longer follow-up periods to improve the quality of the available evidence and further establish its long-term efficacy and clinical utility.

Funnel plots for publication bias (Figure 6) were generally symmetric across most cognitive outcomes, although mild asymmetry was observed for ADAS-Cog. This asymmetry may reflect small-study effects, particularly among rTMS trials, as well as heterogeneity in stimulation parameters and study designs. For emerging modalities such as TPS, TES, and TUS, the assessment of publication bias was also limited by the small number of available controlled trials. Collectively, these limitations indicate that SUCRA rankings reflect the relative position of each intervention within the current evidence network rather than definitive evidence of clinical superiority. Accordingly, differences in treatment rankings should be interpreted in the context of the number of contributing studies, their methodological quality, and the consistency of the available evidence.

Beyond the cognitive outcomes discussed above, ADL represent another clinically relevant outcome in AD neuromodulation. As shown in the NMA forest plot (Figure 4e) and cumulative SUCRA ranking curve (Figure 5e), none of the NMTs showed a statistically significant advantage over Sham for ADCS-ADL. Nevertheless, the SUCRA rankings suggested potentially favorable functional outcomes with TES, rTMS, and tACS. TES may represent a low-risk adjunctive option for supporting functional status because of its favorable safety profile, ease of application, and low caregiver burden, although previous research found no significant cognitive or behavioral benefits of TES monotherapy in patients with AD (van Dijk et al., 2005).

Subgroup analysis further suggested that treatment duration may contribute to heterogeneity in ADCS-ADL outcomes. A 14-day TPS protocol was not associated with a statistically significant effect on daily functioning, whereas longer-duration TPS was associated with poorer ADCS-ADL outcomes. However, this finding should be considered exploratory because the subgroup analysis was based on a small number of studies and participants and requires confirmation in adequately powered multicenter RCTs. The favorable SUCRA ranking of TES for ADCS-ADL also warrants particular caution because only one eligible RCT contributed comparative data. This resulted in sparse network evidence and substantial imprecision in the effect estimate, making it difficult to distinguish treatment effects from study-specific influences or random variation. Moreover, formal assessment of between-study heterogeneity and small-study effects was not feasible. Therefore, the high SUCRA ranking of TES should be considered exploratory rather than interpreted as definitive evidence of clinical benefit.

In addition to cognitive and functional outcomes, neuropsychiatric symptoms remain an important therapeutic target in AD. With respect to neuropsychiatric outcomes, none of the NMTs demonstrated statistically significant improvements in NPI or GDS compared with Sham. Nevertheless, the SUCRA rankings suggested potentially favorable effects of tACS for GDS and TPS for NPI. However, neither modality demonstrated statistically significant superiority over Sham. These findings are consistent with the heterogeneous evidence reported in previous studies, indicating that the effects of NMTs on neuropsychiatric symptoms remain uncertain. For example, Wu et al. (2022). Reported reductions in emotional and behavioral symptoms following TPS, although the between-group differences were not statistically significant. Similarly, Lee et al. (2016) found that rTMS combined with cognitive training did not produce significant antidepressant effects in patients with mild-to-moderate AD, suggesting that placebo effects or other nonspecific factors may have contributed to the observed clinical improvements.

Nevertheless, longer-term follow-up evidence suggests that sustained neuromodulation may provide additional benefits. Notably, Koch et al. (2025) reported that 52 weeks of precuneus-targeted rTMS delayed the progression of neuropsychiatric symptoms, particularly apathy, appetite disturbances, and euphoria. In the present study, subgroup analysis indicated that heterogeneity in NPI outcomes was partly associated with differences in rTMS stimulation frequency. Specifically, 10 Hz rTMS was associated with significantly worse neuropsychiatric outcomes, whereas 20 Hz rTMS and alternating 1/10 Hz stimulation showed non-significant trends toward symptom improvement. These inconsistent findings may reflect small sample sizes, heterogeneity in stimulation protocols, and differences in outcome assessment methods. Future studies should further investigate stimulation frequency as a potential moderator of neuropsychiatric outcomes. The use of standardized stimulation protocols and unified outcome measures may improve between-study comparability and strengthen the reliability of treatment effect estimates.

### 5.3 Core technical mechanisms and clinical application value

The favorable cognitive ranking of TPS observed in Figures 4 and 5 may be partially explained by its capacity to deliver non-invasive mechanical stimulation to relatively deep brain regions involved in memory processing (Zhang et al., 2023). Beisteiner et al. (2020) reported sustained improvements in memory and language function for up to 3 months following TPS in patients with AD, while fMRI findings suggested that these effects may be associated with modulation of memory-related neural networks. Supporting these observations, previous neuroimaging studies have suggested that TPS enhances functional connectivity within hippocampal–parietal memory networks, which may partly underlie the improvements in cognitive outcomes observed across ADAS-Cog, MoCA, and MMSE. Modulation of connectivity between the ventromedial and salience networks has also been proposed as a potential mechanism underlying its effects on depressive symptoms reflected by GDS scores (Chen et al., 2024). In addition, Cont et al. (2022) reported that TPS was safe and well tolerated across patients with mild-to-severe AD and was associated with short-term improvements in cognition and depressive symptoms. Nevertheless, these mechanistic interpretations remain preliminary and are supported by limited evidence. Future studies integrating neuroimaging biomarkers with standardized clinical outcome measures are needed to further clarify the mechanisms through which TPS influences cognitive and neuropsychiatric outcomes.

The cognitive benefits of rTMS observed in this NMA may be related to its capacity to modulate cortical excitability, neural activity, and synaptic plasticity through pulsed magnetic stimulation (Bai et al., 2018; Jafari et al., 2020; Wang et al., 2020; Guan et al., 2022). HF-rTMS has been associated with enhanced long-term potentiation (LTP) and corresponding improvements in cognitive performance (Li et al., 2021), while stimulation of the left parietal cortex may be particularly beneficial for memory in patients with mild-to-moderate AD (Jia et al., 2021). Beyond these neurophysiological effects, rTMS may also modulate AD-related pathological processes, as reductions in circulating Aβ1-40 and p-tau181 have been reported following treatment (Wagemann et al., 2024). These findings provide a potential biological basis for the cognitive benefits observed with rTMS in the present analysis, although the relative contributions of altered cortical excitability, synaptic plasticity, and pathological modulation remain to be clarified.

tDCS influences neuronal excitability by modifying the resting membrane potential (Truong and Bikson, 2018). Emerging evidence suggests that its effects in AD may vary according to stimulation target and disease stage. Temporoparietal stimulation (1.5 mA; 15 min/session) augmented word recognition memory, while recurrent bilateral temporal stimulation (20 min/session; 10 sessions) improved overall cognitive function. The latter was also linked to elevated blood Aβ1–42 levels, with cognitive enhancement correlating with changes in Aβ1–42, suggesting a potential relationship (Ferrucci et al., 2008; Khedr et al., 2019). Conversely, The limited cognitive benefits of frontotemporal tDCS in advanced AD may be partly attributable to reduced cortical responsiveness associated with disease progression (Bystad et al., 2016).

tACS administers biphasic sinusoidal alternating current to the scalp and may enhance cognitive function by modulating synaptic plasticity, the release of cognition-related neurotransmitters, and brain network connections (Hone-Blanchet et al., 2016; Palop and Mucke, 2016; Tavakoli and Yun, 2017; Meyer et al., 2019; Elyamany et al., 2021). In patients with AD, these effects may be reflected in improvements in episodic memory, particularly improved language recall after stimulation of the medial parietal cortex and precuneus, as well as increased episodic memory scores after treatment (Benussi et al., 2021; Benussi et al., 2022). Furthermore, tACS in conjunction with cognitive training has demonstrated greater efficacy than cognitive training alone, as evidenced by improved performance on memory assessments such as the Wechsler Memory Scale (Kehler et al., 2020), with similar synergistic effects on overall cognitive function reported in other studies (Moussavi et al., 2021).

As illustrated in Figures 4 and 5, PBM may represent a promising non-pharmacological intervention for AD. Its potential neuroprotective and cognitive effects may involve reducing Aβ and tau-related pathology, restoring mitochondrial function, suppressing neuroinflammation, and enhancing antioxidant defenses and BDNF-dependent synaptic pathways (Enengl et al., 2020; Abijo et al., 2023). Early clinical evidence suggests that PBM is well tolerated and may provide cognitive benefits across different stages of disease. In mild-to-moderate AD, combined cranial and abdominal PBM was associated with favorable safety and preliminary cognitive improvement (Blivet et al., 2022),while in MCI, improvements in visual memory accompanied by attenuated task-related hemodynamic responses may reflect enhanced neural efficiency (Chan et al., 2021).

TUS provides relatively high spatial resolution and may facilitate precise targeting of deep brain regions. In a study of 60 individuals with AD, TUS showed a non-significant trend toward improved global cognitive performance, while improvements in depressive symptoms, activation of memory-related brain regions, and functional connectivity within the dorsal attention network were also observed (Beisteiner et al., 2020). These preliminary findings suggest that the potential effects of TUS may extend beyond global cognition to neuropsychiatric symptoms and brain network modulation; Overall, the present findings, together with existing evidence, suggest that PBM and TUS may represent promising adjunctive neuromodulation approaches for AD, although their relative therapeutic value remains to be confirmed.

## 6 Limitations

This study has several limitations. First, the evidence base for several interventions was limited, with some modalities, such as DBS and TES, supported by only one RCT and TPS and TUS by relatively few studies. This sparse evidence may reduce the precision and robustness of efficacy estimates and warrants cautious interpretation of their treatment rankings. Second, although NMA allows indirect comparisons among neuromodulation techniques, substantial variability existed in stimulation parameters, including frequency, intensity, duration, treatment schedules, and target regions. Such heterogeneity may have contributed to differences in treatment response and reduced comparability across interventions. Third, the methodological quality of the included studies may also have introduced uncertainty into the findings. Approximately 25% of studies showed some concerns and 8.3% were rated as having a high risk of bias, mainly related to selective reporting and outcome measurement. Fourth, most included studies were conducted in Asian populations, potentially limiting the generalizability of the findings because of differences in genetic background, education level, healthcare systems, and rehabilitation practices. In addition, variability in baseline disease severity and the limited sensitivity of global cognitive scales (e.g., MMSE and MoCA) to domain-specific impairments may have affected the detection of treatment effects. The inclusion of only English-language studies may also have introduced language bias. Furthermore, most studies evaluated single neuromodulation modalities, limiting the assessment of potential synergistic effects of combined interventions. Finally, most included trials reported only short-term outcomes, and the lack of long-term follow-up limits the evaluation of sustained treatment effects. Future international multicenter RCTs should therefore use standardized stimulation protocols, recruit larger and more diverse populations, incorporate longer follow-up, and integrate neuroimaging and electrophysiological biomarkers (e.g., EEG and fMRI) to improve the reliability of treatment estimates and facilitate mechanism-based and personalized neuromodulation strategies.

## 7 Conclusion

This NMA confirms that neuromodulation enhances cognitive functioning in patients with AD. Specifically, TPS demonstrates potential advantages in improving overall cognitive function; rTMS has a statistically significant effect on improving ADAS-Cog scores; TES, rTMS, and tACS may benefit activities of daily living; and TPS and tACS show more favorable trends in improving neuropsychiatric symptoms. Overall, these findings support outcome-specific rather than universally superior neuromodulation strategies and highlight the need for individualized treatment selection supported by future multicenter trials.

## Data Availability

The original contributions presented in the study are included in the article/Supplementary material, further inquiries can be directed to the corresponding author.
