# Supplementary material for: Comparative efficacy of neuromodulation techniques for Alzheimer’s disease: a systematic review and network meta-analysis of randomized controlled trials
Source: Front Neurosci. 2026 Sep 11;20:1890235. doi: 10.3389/fnins.2026.1890235 (PMC13612472; doi:10.3389/fnins.2026.1890235)

Pairwise Comparison Forest Plot

a

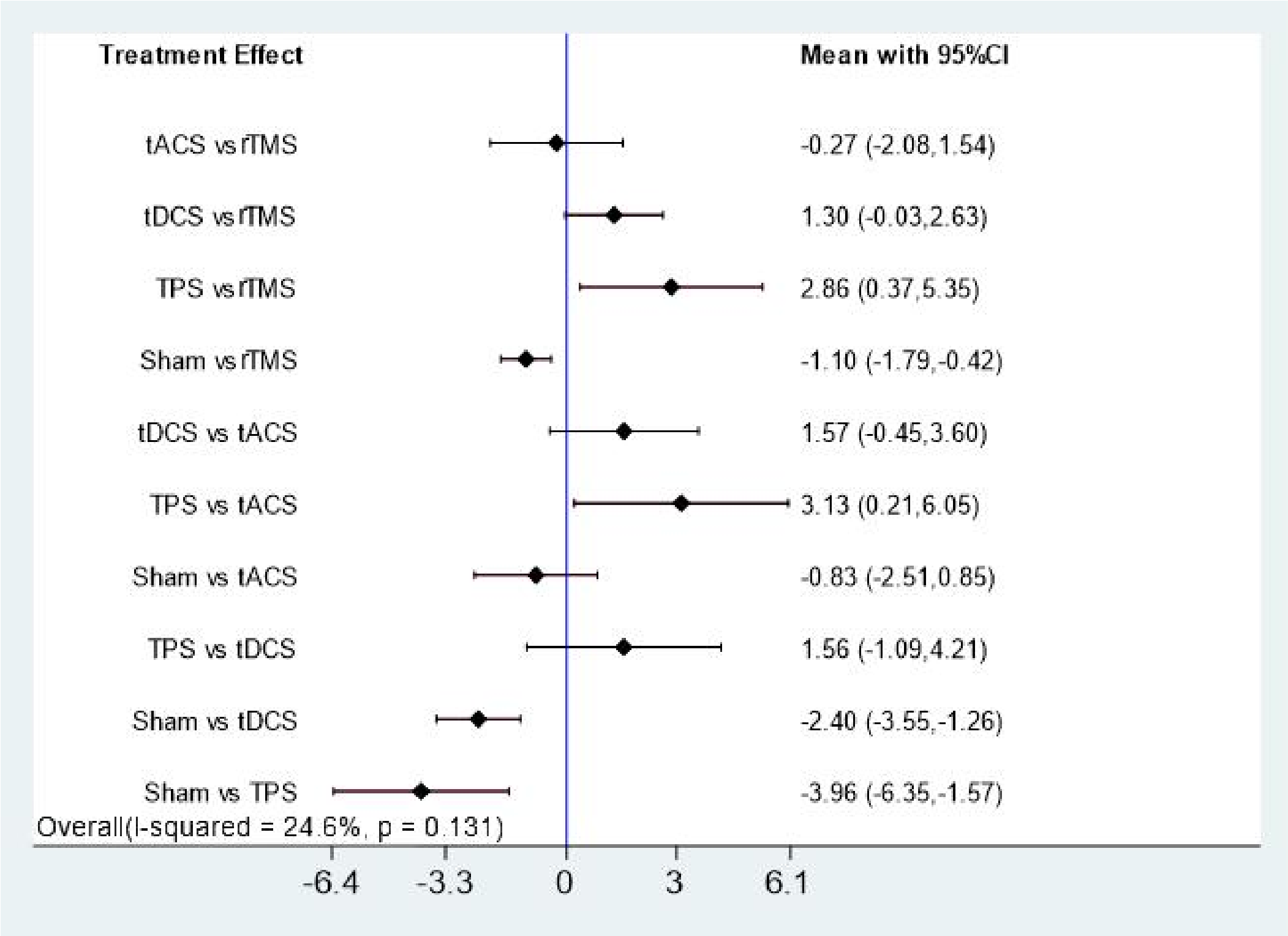

b

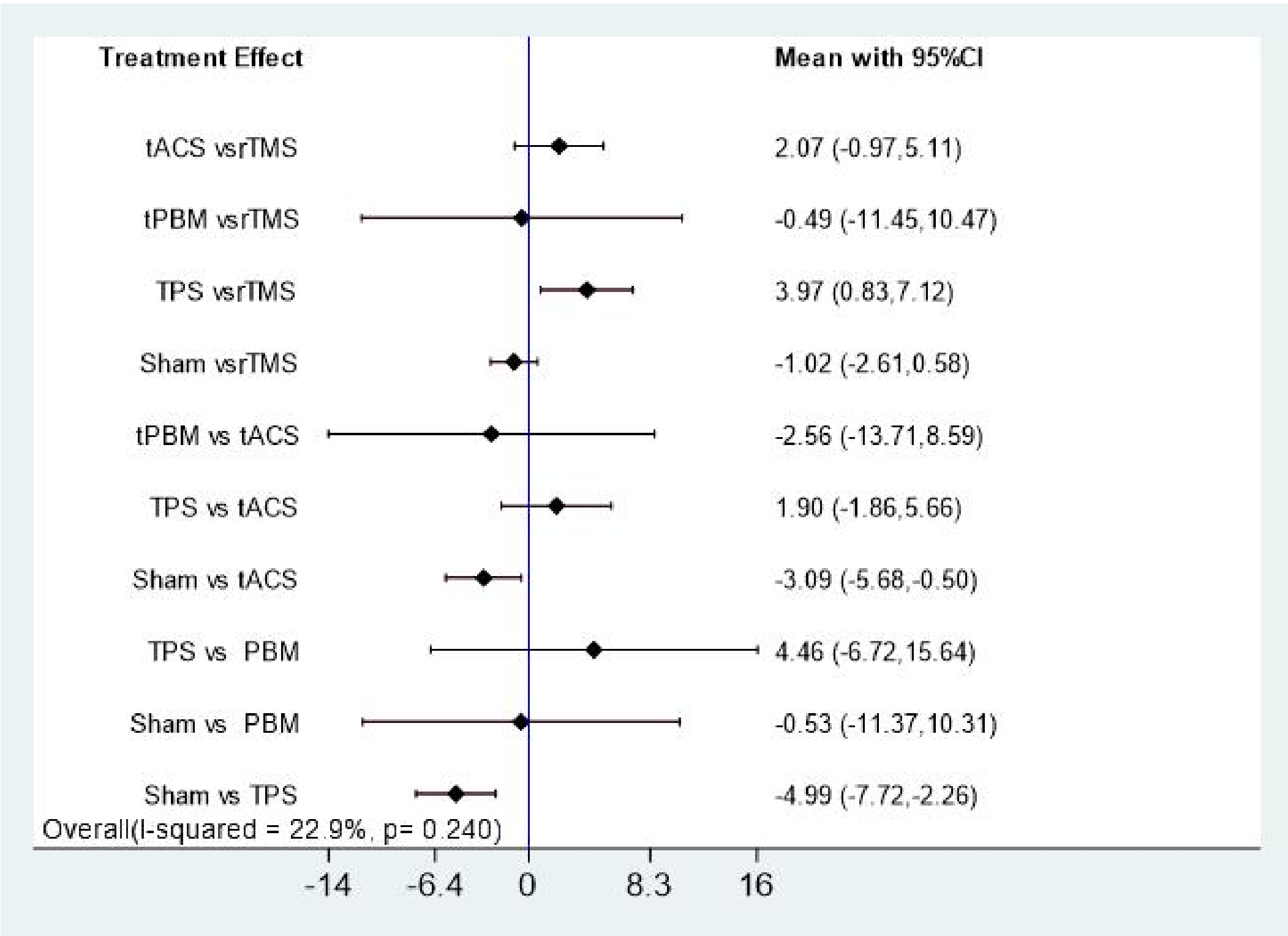

c

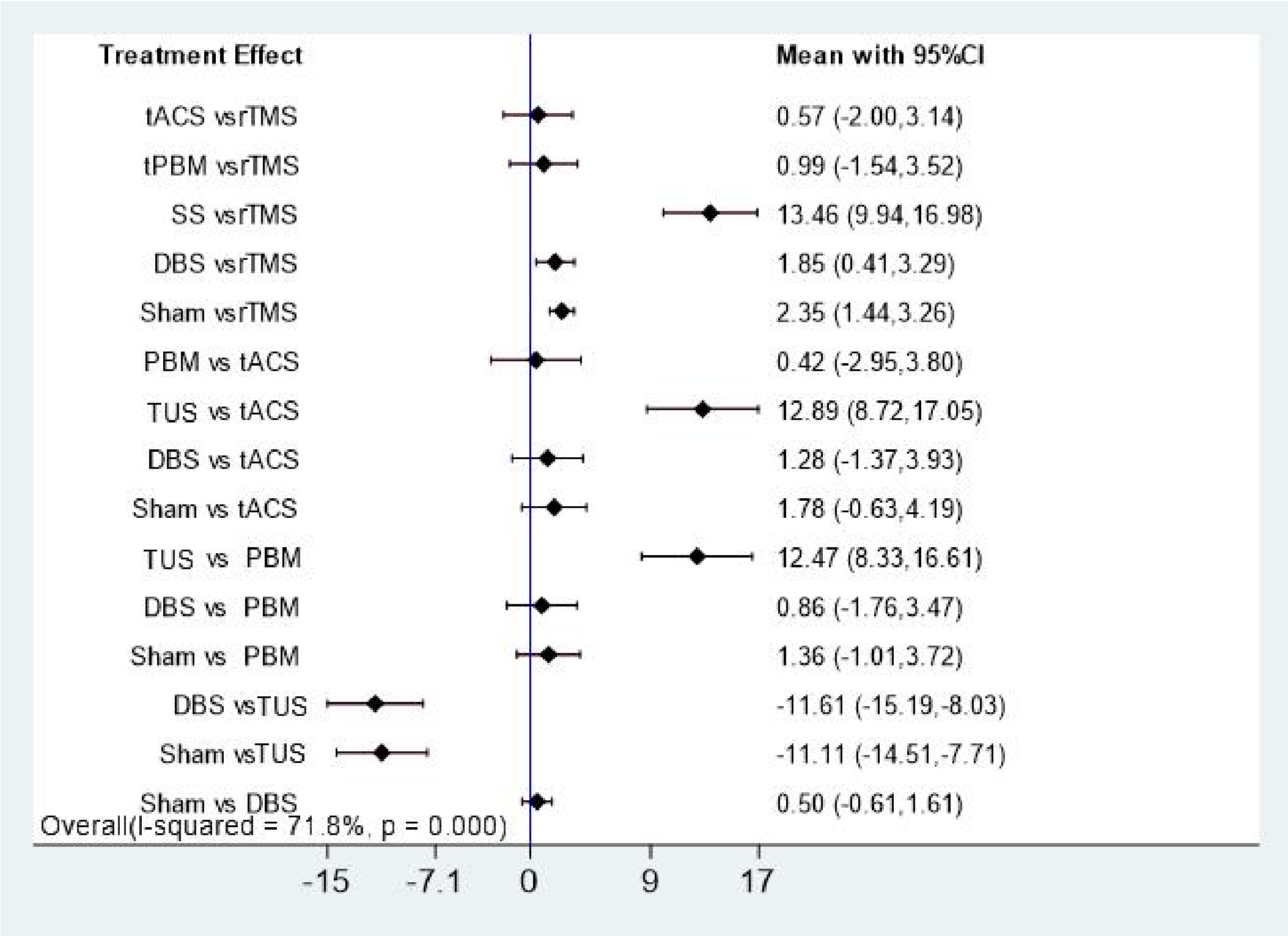
Treatment Effect
Mean with 95%CI
tACS vsrTMS
0.57 (-2.00,3.14)
tPBM vsrTMS
0.99 (-1.54,3.52)
SS vsrTMS
13.46 (9.94,16.98)
DBS vsrTMS
1.85 (0.41,3.29)
Sham vsrTMS
2.35 (1.44,3.26)
PBM vs tACS
0.42 (-2.95,3.80)
TUS vs tACS
12.89 (8.72,17.05)
DBS vs tACS
1.28 (-1.37,3.93)
Sham vs tACS
1.78 (-0.63,4.19)
TUS vs PBM
12.47 (8.33,16.61)
DBS vs PBM
0.86 (-1.76,3.47)
Sham vs PBM
1.36 (-1.01,3.72)
DBS vsTUS
-11.61 (-15.19,-8.03)
Sham vsTUS
-11.11 (-14.51,-7.71)
Sham vs DBS
0.50 (-0.61,1.61)
Overall(I-squared = 71.8%, p = 0.000)
-15
-7.1
0
9
17

d

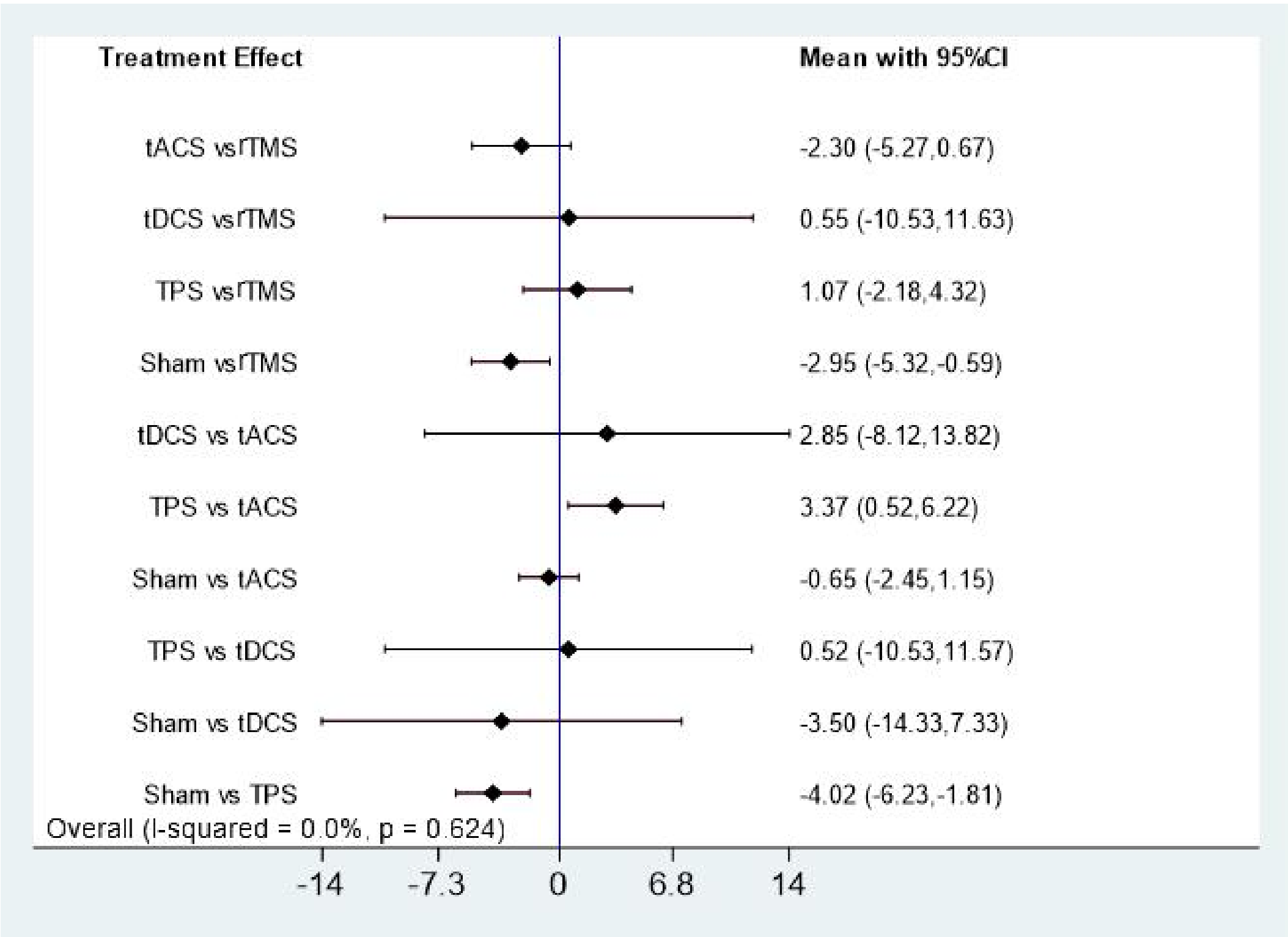
Treatment Effect
Mean with 95%CI
tACS vsrTMS
-2.30 (-5.27,0.67)
tDCS vsrTMS
0.55 (-10.53,11.63)
TPS vsrTMS
1.07 (-2.18,4.32)
Sham vsrTMS
-2.95 (-5.32,-0.59)
tDCS vs tACS
2.85 (-8.12,13.82)
TPS vs tACS
3.37 (0.52,6.22)
Sham vs tACS
-0.65 (-2.45,1.15)
TPS vs tDCS
0.52 (-10.53,11.57)
Sham vs tDCS
-3.50 (-14.33,7.33)
Sham vs TPS
-4.02 (-6.23,-1.81)
Overall (I-squared = 0.0%, p = 0.624)
-14
-7.3
0
6.8
14

e

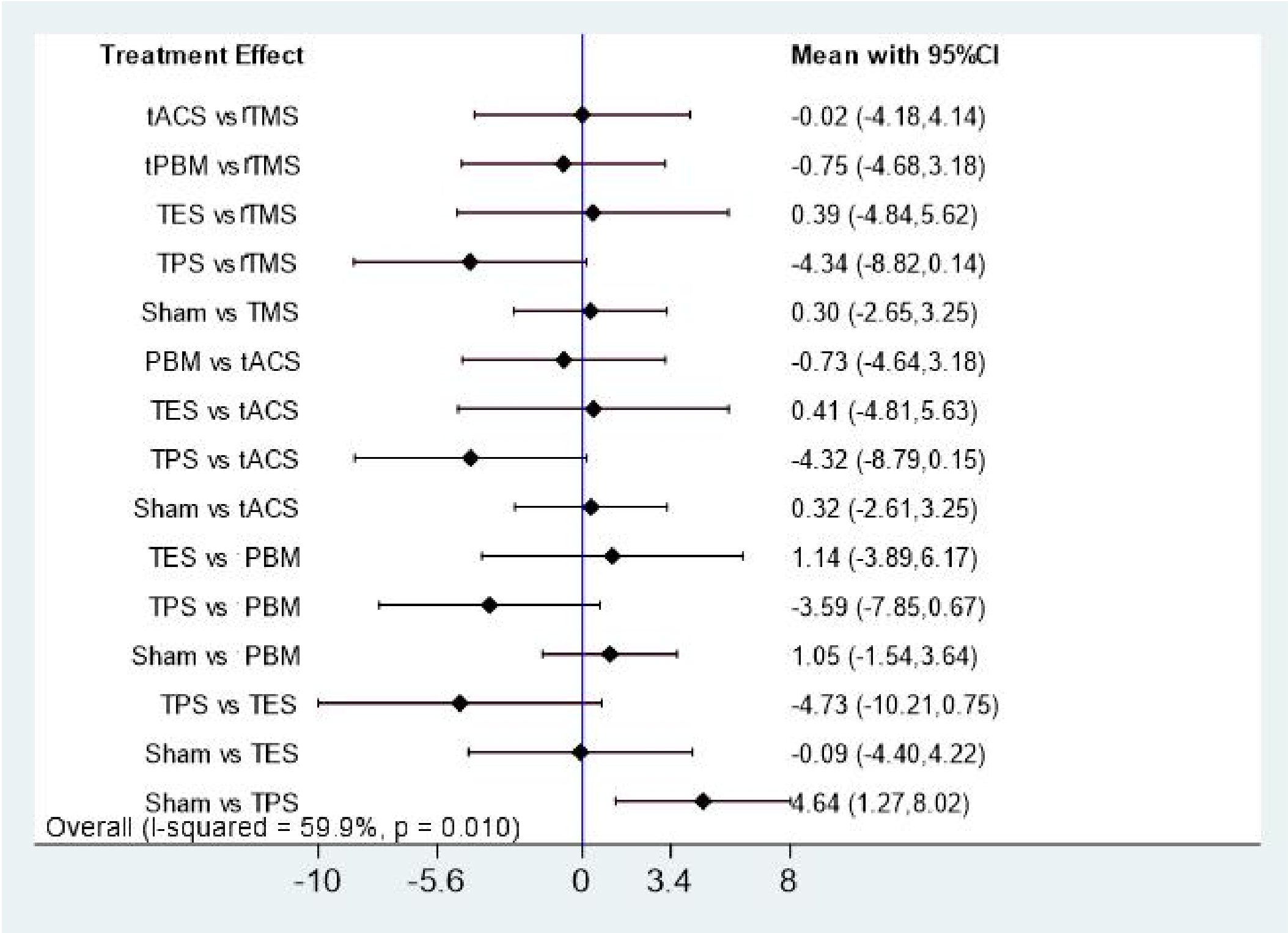
Treatment Effect
Mean with 95%CI
tACS vs rTMS
-0.02 (-4.18,4.14)
tPBM vs rTMS
-0.75 (-4.68,3.18)
TES vs rTMS
0.39 (-4.84,5.62)
TPS vs rTMS
-4.34 (-8.82,0.14)
Sham vs TMS
0.30 (-2.65,3.25)
PBM vs tACS
-0.73 (-4.64,3.18)
TES vs tACS
0.41 (-4.81,5.63)
TPS vs tACS
-4.32 (-8.79,0.15)
Sham vs tACS
0.32 (-2.61,3.25)
TES vs PBM
1.14 (-3.89,6.17)
TPS vs PBM
-3.59 (-7.85,0.67)
Sham vs PBM
1.05 (-1.54,3.64)
TPS vs TES
-4.73 (-10.21,0.75)
Sham vs TES
-0.09 (-4.40,4.22)
Sham vs TPS
4.64 (1.27,8.02)
Overall (I-squared = 59.9%, p = 0.010)
-10
-5.6
0
3.4
8

f

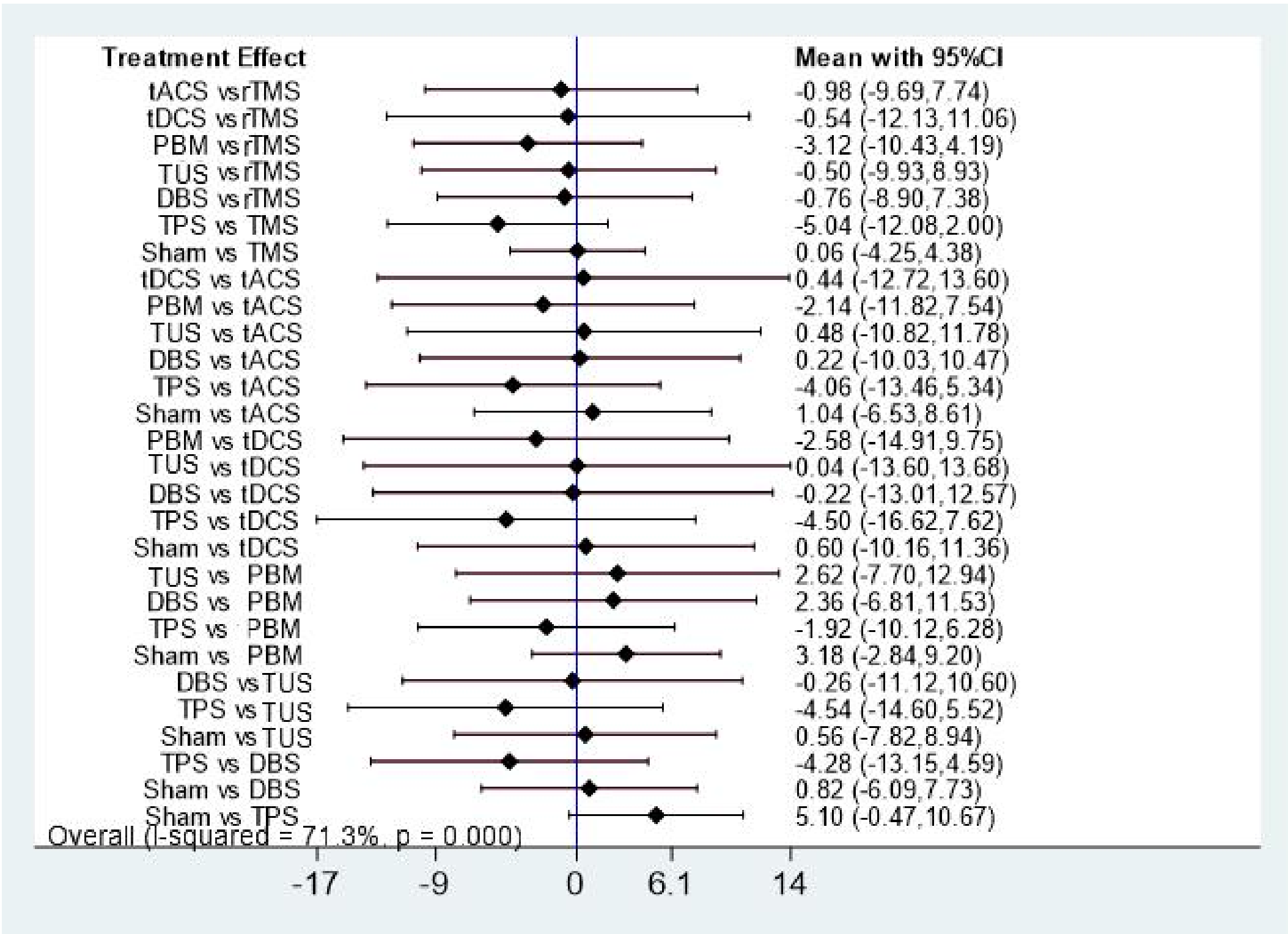
Treatment Effect
Mean with 95%CI
tACS vs rTMS
-0.98 (-9.69,7.74)
tDCS vs rTMS
-0.54 (-12.13,11.06)
PBM vs rTMS
-3.12 (-10.43,4.19)
TUS vs rTMS
-0.50 (-9.93,8.93)
DBS vs rTMS
-0.76 (-8.90,7.38)
TPS vs TMS
-5.04 (-12.08,2.00)
Sham vs TMS
0.06 (-4.25,4.38)
tDCS vs tACS
0.44 (-12.72,13.60)
PBM vs tACS
-2.14 (-11.82,7.54)
TUS vs tACS
0.48 (-10.82,11.78)
DBS vs tACS
0.22 (-10.03,10.47)
TPS vs tACS
-4.06 (-13.46,5.34)
Sham vs tACS
1.04 (-6.53,8.61)
PBM vs tDCS
-2.58 (-14.91,9.75)
TUS vs tDCS
0.04 (-13.60,13.68)
DBS vs tDCS
-0.22 (-13.01,12.57)
TPS vs tDCS
-4.50 (-16.62,7.62)
Sham vs tDCS
0.60 (-10.16,11.36)
TUS vs PBM
2.62 (-7.70,12.94)
DBS vs PBM
2.36 (-6.81,11.53)
TPS vs PBM
-1.92 (-10.12,6.28)
Sham vs PBM
3.18 (-2.84,9.20)
DBS vs TUS
-0.26 (-11.12,10.60)
TPS vs TUS
-4.54 (-14.60,5.52)
Sham vs TUS
0.56 (-7.82,8.94)
TPS vs DBS
-4.28 (-13.15,4.59)
Sham vs DBS
0.82 (-6.09,7.73)
Sham vs TPS
5.10 (-0.47,10.67)
Overall (I-squared = 71.3%, p = 0.000)
-17
-9
0
6.1
14

g

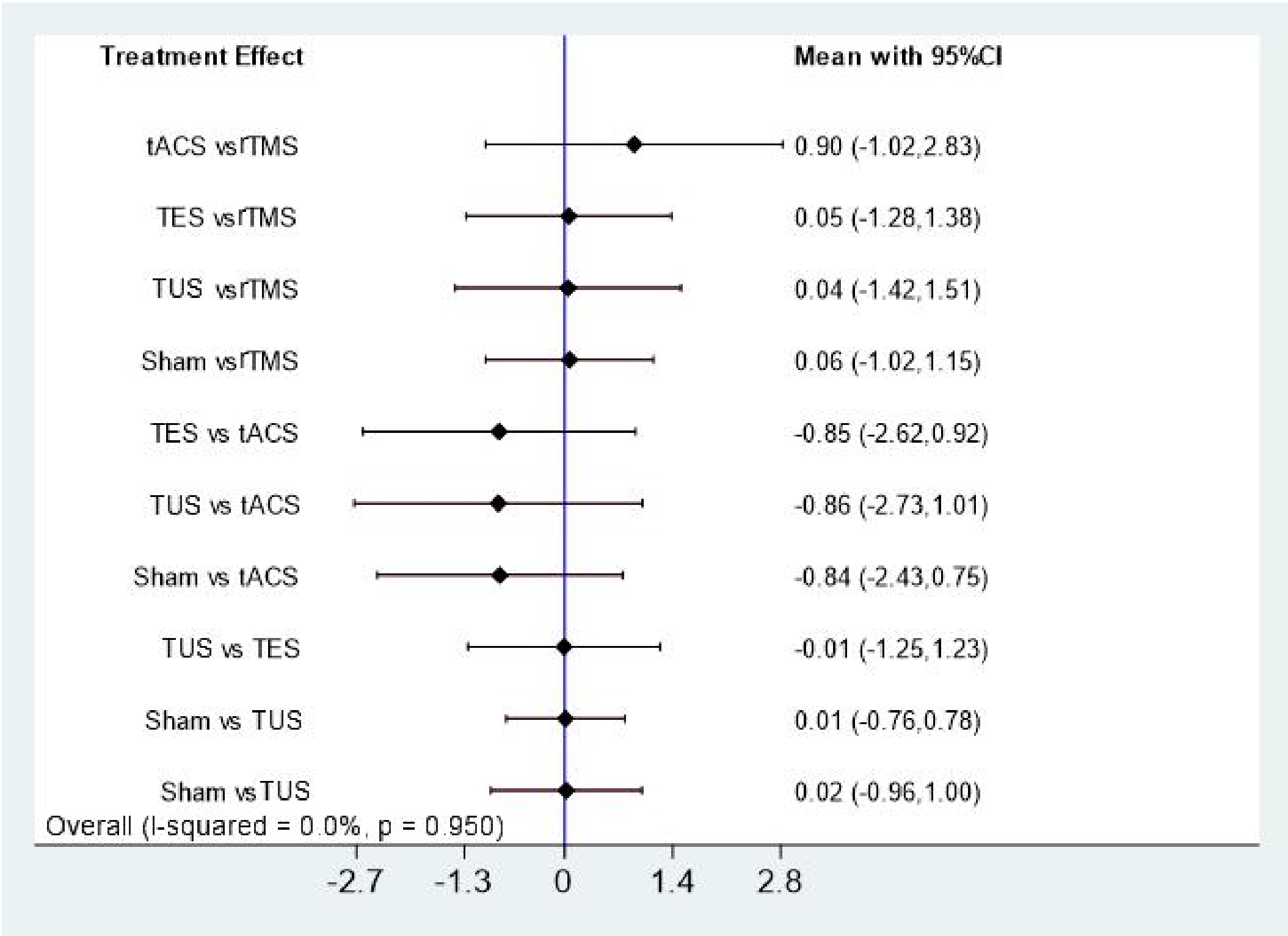

(a) Mini-Mental State Examination (MMSE), (b) Montreal Cognitive Assessment (MoCA), (c) Alzheimer’s Disease Assessment Scale-Cognitive Subscale (ADAS-Cog), (d) Alzheimer’s Disease Cooperative Study-Activities of Daily Living (ADCS-ADL), (e)Boston Naming Test (BNT), (f) Neuropsychiatric Inventory (NPI), (g) Geriatric Depression Scale (GDS).

Heterogeneity Test

Alzheimer’s Disease Cooperative Study-Activities of Daily Living (ADCS-ADL)

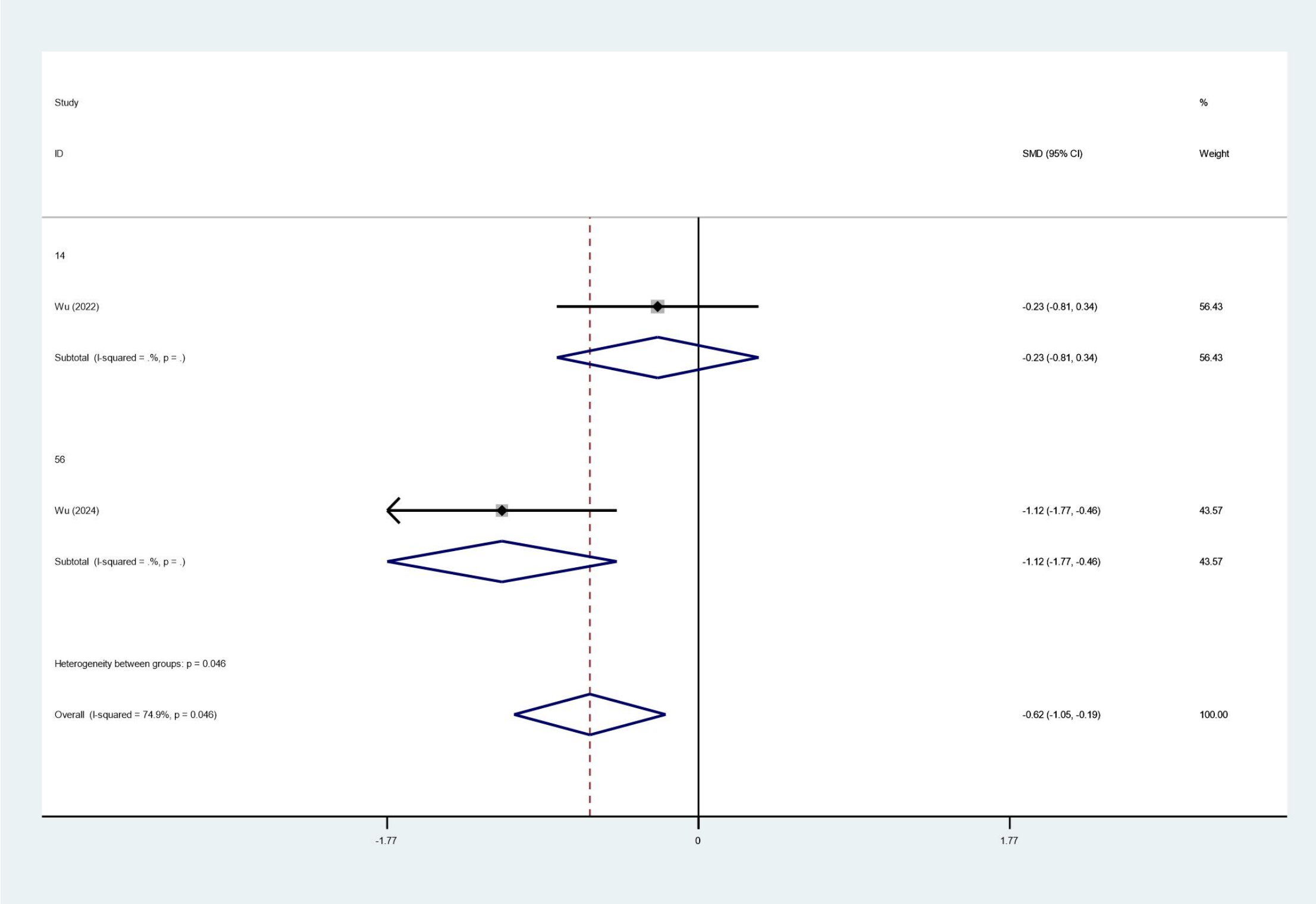

Neuropsychiatric Inventory (NPI)

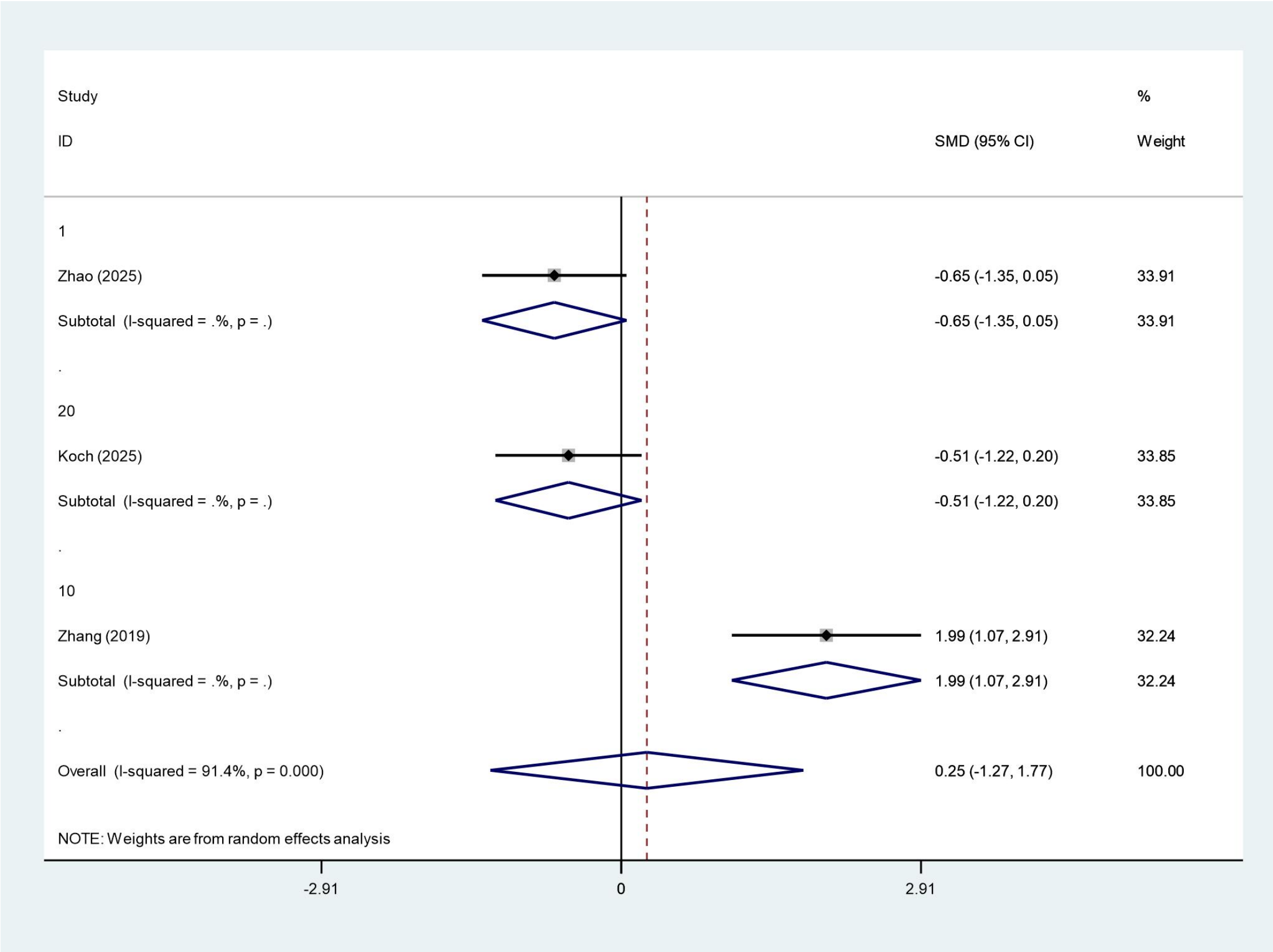

Alzheimer’ s Disease Assessment Scale-Cognitive Subscale (ADAS-Cog)

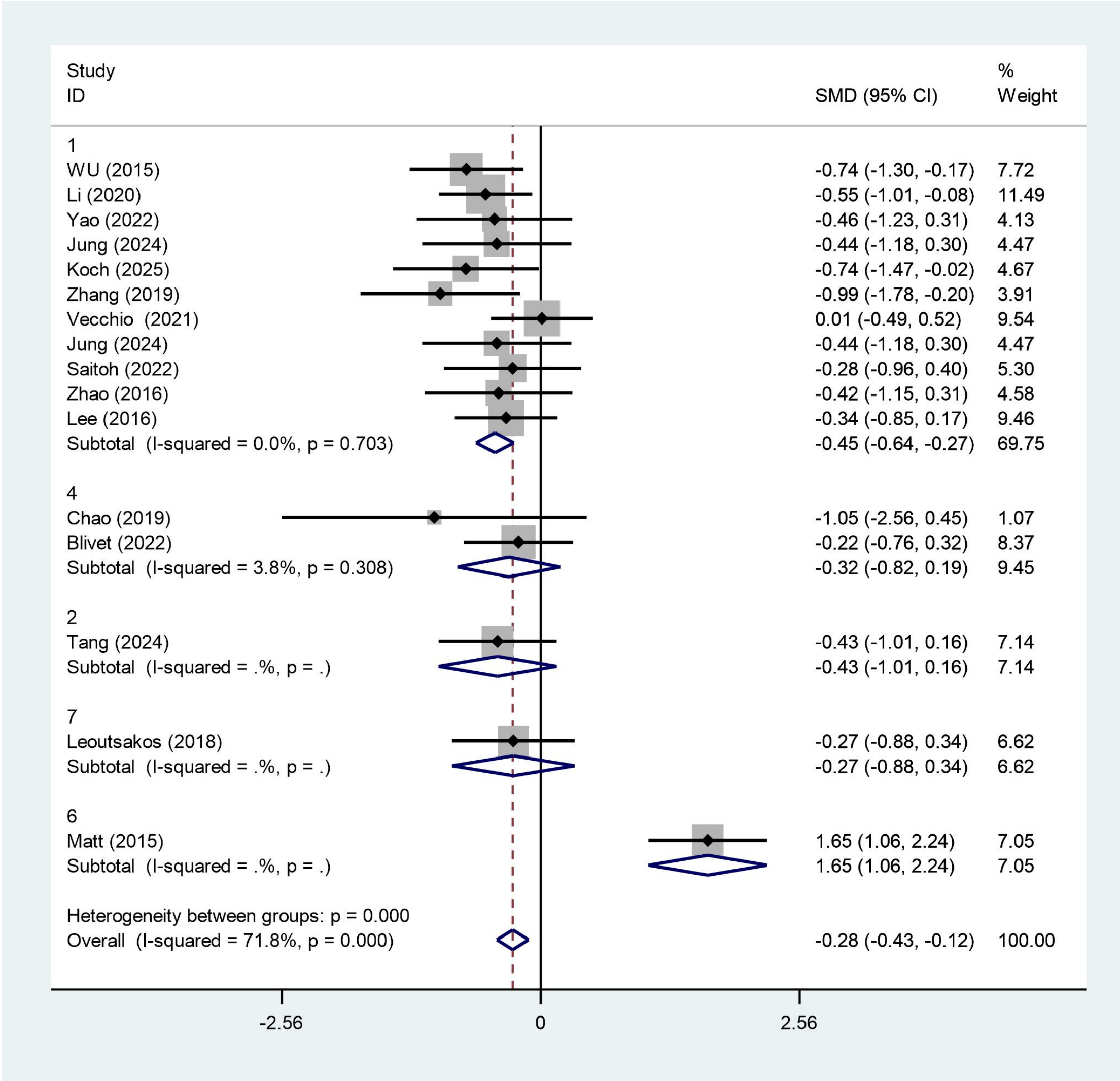

Supplement: Supplementary file 1 [file Data_Sheet_1.PDF]
